# Preliminary findings on long‐term effects of fMRI neurofeedback training on functional networks involved in sustained attention

**DOI:** 10.1002/brb3.3217

**Published:** 2023-08-18

**Authors:** Gustavo Santo Pedro Pamplona, Jennifer Heldner, Robert Langner, Yury Koush, Lars Michels, Silvio Ionta, Carlos Ernesto Garrido Salmon, Frank Scharnowski

**Affiliations:** ^1^ Sensory‐Motor Laboratory (SeMoLa), Jules‐Gonin Eye Hospital/Fondation Asile des Aveugles Department of Ophthalmology/University of Lausanne Lausanne Switzerland; ^2^ InBrain Lab, Department of Physics University of Sao Paulo Ribeirao Preto Brazil; ^3^ Department of Psychiatry, Psychotherapy and Psychosomatics, Psychiatric Hospital University of Zurich Zurich Switzerland; ^4^ Rehabilitation Engineering Laboratory (RELab), Department of Health Sciences and Technology ETH Zurich Zurich Switzerland; ^5^ Institute of Systems Neuroscience Heinrich Heine University Dusseldorf Dusseldorf Germany; ^6^ Institute of Neuroscience and Medicine, Brain & Behaviour (INM‐7) Research Centre Julich Julich Germany; ^7^ Department of Radiology and Biomedical Imaging, Yale School of Medicine Yale University New Haven Connecticut USA; ^8^ Department of Neuroradiology University Hospital Zurich Zurich Switzerland; ^9^ Neuroscience Center Zurich University of Zurich and Swiss Federal Institute of Technology Zurich Switzerland; ^10^ Department of Cognition, Emotion, and Methods in Psychology, Faculty of Psychology University of Vienna Vienna Austria

**Keywords:** fMRI‐neurofeedback, follow‐up, functional connectivity, sustained attention

## Abstract

**Introduction:**

Neurofeedback based on functional magnetic resonance imaging allows for learning voluntary control over one's own brain activity, aiming to enhance cognition and clinical symptoms. We previously reported improved sustained attention temporarily by training healthy participants to up‐regulate the differential activity of the sustained attention network minus the default mode network (DMN). However, the long‐term brain and behavioral effects of this training have not yet been studied. In general, despite their relevance, long‐term learning effects of neurofeedback training remain under‐explored.

**Methods:**

Here, we complement our previously reported results by evaluating the neurofeedback training effects on functional networks involved in sustained attention and by assessing behavioral and brain measures before, after, and 2 months after training. The behavioral measures include task as well as questionnaire scores, and the brain measures include activity and connectivity during self‐regulation runs without feedback (i.e., transfer runs) and during resting‐state runs from 15 healthy individuals.

**Results:**

Neurally, we found that participants maintained their ability to control the differential activity during follow‐up sessions. Further, exploratory analyses showed that the training increased the functional connectivity between the DMN and the occipital gyrus, which was maintained during follow‐up transfer runs but not during follow‐up resting‐state runs. Behaviorally, we found that enhanced sustained attention right after training returned to baseline level during follow‐up.

**Conclusion:**

The discrepancy between lasting regulation‐related brain changes but transient behavioral and resting‐state effects raises the question of how neural changes induced by neurofeedback training translate to potential behavioral improvements. Since neurofeedback directly targets brain measures to indirectly improve behavior in the long term, a better understanding of the brain–behavior associations during and after neurofeedback training is needed to develop its full potential as a promising scientific and clinical tool.

## Graphical abstract

We present what we believe to be the first study showing that individuals are able to maintain learned self‐regulation of large‐scale brain networks months after the end of a neurofeedback training based on functional magnetic resonance imaging. We also observed that the functional connectivity of the trained regions changed immediately after training and months later, both during active self‐regulation and during resting‐state. Despite the lasting self‐regulation ability and brain changes due to neurofeedback, we observed that the behavioral changes did not persist months after the end of training.

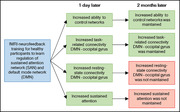

## 1 INTRODUCTION

Neurofeedback is a form of biofeedback that provides individuals with real‐time sensory information from their own brain activity, over which voluntary control can be learned with training (Sitaram et al., 2017). Neurofeedback training has been associated with behavioral changes, which makes it an interesting approach for studying brain–behavior relationships (Sitaram et al., 2017; Sulzer, Haller et al., 2013). Neurofeedback training has also produced clinical benefits, which makes it a promising clinical intervention for the treatment of neurological and psychiatric disorders (e.g., Linhartová et al., 2019; Martz et al., 2020; Sokunbi, 2017; Sulzer, Haller et al., 2013; Taschereau‐Dumouchel et al., 2022; Wang et al., 2018). The reported effects of neurofeedback training include transient as well as lasting changes. For cognitive enhancement and clinical applications of neurofeedback training, lasting effects are particularly important. Behaviorally, several studies reported that neurofeedback was associated with changes lasting beyond the initial training (Amano et al., 2016; Karch et al., 2022; Kohl et al., 2019; Mehler et al., 2018; Nicholson et al., 2020; Van Doren et al., 2019; Young et al., 2018; Zilverstand et al., 2015). Some studies even found that clinical symptoms “continue to improve for weeks after neurofeedback” training (Rance et al., 2018). Also neurally, lasting plastic brain changes have been reported, including resting‐state functional connectivity (FC; Megumi et al., 2015; Nicholson et al., 2020; Scheinost et al., 2013; Young et al., 2018; Yuan et al., 2014; G. Zhang et al., 2013) and brain structural changes (Marins et al., 2019; Sampaio‐Baptista et al., 2021). Lasting brain changes combined with behavioral modulations induced by neurofeedback training provide new insights into how they relate to each other. Hence, investigating long‐term effects of neurofeedback training helps understanding its learning mechanisms and might facilitate the use of neurofeedback for enhancing cognition and clinical symptoms.

Here, we investigate lasting behavioral and neural changes following our previously described neurofeedback training of sustained attention (Pamplona, Heldner et al., 2020). While the previously published results focus on how control over the sustained attention network (SAN) and the default mode network (DMN) is learned and on immediate behavioral effects, the current study focuses on persisting connectivity and behavioral changes related to sustained attention. Sustained attention is a cognitive function that supports the continuous focus on a particular external object for extended periods of time. Neuroimaging correlates of sustained attention comprise the SAN (Langner & Eickhoff, 2013), which combines regions from the frontoparietal control network (FPCN; Dosenbach et al., 2008) and the dorsal attention network (DAN; Yeo et al., 2011). In contrast, DMN activation is related to internally focused cognitive processes and mind‐wandering (Andrews‐Hanna et al., 2014; Raichle et al., 2001). DMN activation is therefore associated with stimulus‐independent thoughts and reduced attention during the execution of an externally oriented task (Hinds et al., 2013; Lawrence et al., 2003; Thompson et al., 2013; Weissman et al., 2006). The SAN (more specifically, its DAN components; Spreng, 2012) and DMN are intrinsically anticorrelated, as they are engaged in antagonistic processes reflecting externally versus internally oriented attention (Fox et al., 2005; Spreng, 2012). Mounting neuroscientific evidence shows that the duality of external/internal attentional processing is controlled by two other networks, the FPCN and the salience network (SAL) (Andrews‐Hanna et al., 2014; Fox et al., 2005; Smallwood et al., 2012; Spreng, 2012). The FPCN is involved in the top‐control flexible modulation of other brain regions according to the task demand, either externally or internally oriented (Andrews‐Hanna et al., 2014; Dixon et al., 2018). The SAL performs dynamically switching between external and internal networks according to the detection of significant stimuli (Andrews‐Hanna et al., 2014; Seeley et al., 2007). Several studies have shown the feasibility of volitional control of DMN activity using functional magnetic resonance imaging (fMRI)‐neurofeedback, either defined as a single region (Garrison et al., 2013; Harmelech et al., 2015; G. Zhang et al., 2013), multiple regions (McDonald et al., 2017), or through relative DMN activity, compared to other large‐scale networks (Kim et al., 2019; J. Zhang et al., 2023). We recently demonstrated that sustained attention can be improved to some extent through training simultaneous up‐regulation of the SAN and down‐regulation of the DMN using fMRI neurofeedback (Pamplona, Heldner et al., 2020). We found that participants in the neurofeedback group were able to regulate their differential SAN‐DMN activity and showed improved sustained attention directly after the training. A test–retest behavioral control group included in our previously reported study, which only performed the behavioral sustained attention tasks but did not undergo neurofeedback training, showed that the improved sustained attention was not due to practice effects.

Regarding lasting effects, we hypothesized that the neural and behavioral changes induced by our neurofeedback training approach that was previously described (Pamplona, Heldner et al., 2020) would persist beyond the training. Specifically, we hypothesized that regulation performance in brain regions successfully trained with neurofeedback and the associated improved sustained attention would be maintained in the long term. We also hypothesized that lasting FC changes specific to the successfully trained brain regions would be observed. To test these hypotheses, we analyzed fMRI data from runs without feedback (i.e., transfer runs) and resting‐state runs, from before, immediately after, and 2 months after neurofeedback training. We also explored the data in terms of immediate and lasting whole‐brain activation and FC changes. Finally, we explored associations between brain connectivity changes and behavioral effects. All the results derived from brain and behavior data related to lasting changes (i.e., 2 months after neurofeedback training), as well as all results related to FC, are novel and have not been published elsewhere. Specifically, we investigated (i) the persistence of learned regulation in SAN, DMN, and their constituent regions 2 months after training to characterize maintained self‐regulation; (ii) changes in pre‐training FC directly after training and 2 months later to investigate lasting brain connectivity alterations with (transfer runs) and without regulation (resting‐state runs); (iii) changes in resting‐state FC of SAN and DMN regions directly after training and 2 months later using a graph theoretical approach; (iv) persistence of training‐induced attention measured by task and questionnaires 2 months after neurofeedback training to assess the permanence of behavioral effects arising from neurofeedback training; and (v) associations of FC changes in transfer and resting‐state runs with behavioral changes directly after training and 2 months later.

## 2 MATERIALS AND METHODS

### 2.1 Participants

FundRed RegistryWe included data from our previously published study (Pamplona, Heldner et al., 2020), which comprised a neurofeedback training group (referred as “NF group”) performing sustained attention tasks before and after training. The first study also included a behavioral control group of individuals who only performed the attention tasks twice without neurofeedback training (referred as “TR group”), separated by an interval of 7.2 ± 0.4 days, which corresponds to the duration of the neurofeedback training in the experimental group. For the present study, we analyzed data only from the neurofeedback group, which consisted of 15 healthy volunteers (five females, mean age: 27.9 ± 3.3 years old, age range = [22.6, 34.5] years old). Since the behavioral control group did not perform any follow‐up measurements, they were not considered in the current study. Data from the neurofeedback training group included psychometric tasks, transfer runs, and resting‐state runs before, directly after, and 2 months after neurofeedback training. Exclusion criteria were left‐handedness, strong vision deficiency that could not be corrected using contact lenses, insufficient knowledge of English, history of mental and/or cardiovascular disorders, not being able to abstain from alcohol or other drugs during the days of the experiment, and magnetic resonance imaging (MRI) contraindications. This study was approved by the local ethics committee of the Canton of Zurich in Switzerland. All participants read and signed the informed consent in accordance with the Declaration of Helsinki (2013) before taking part in the study. They received financial compensation of 25 CHF per hour for their participation.

### 2.2 Experimental procedure

#### 2.2.1 Timeline of experimental procedure

**FIGURE 1:**
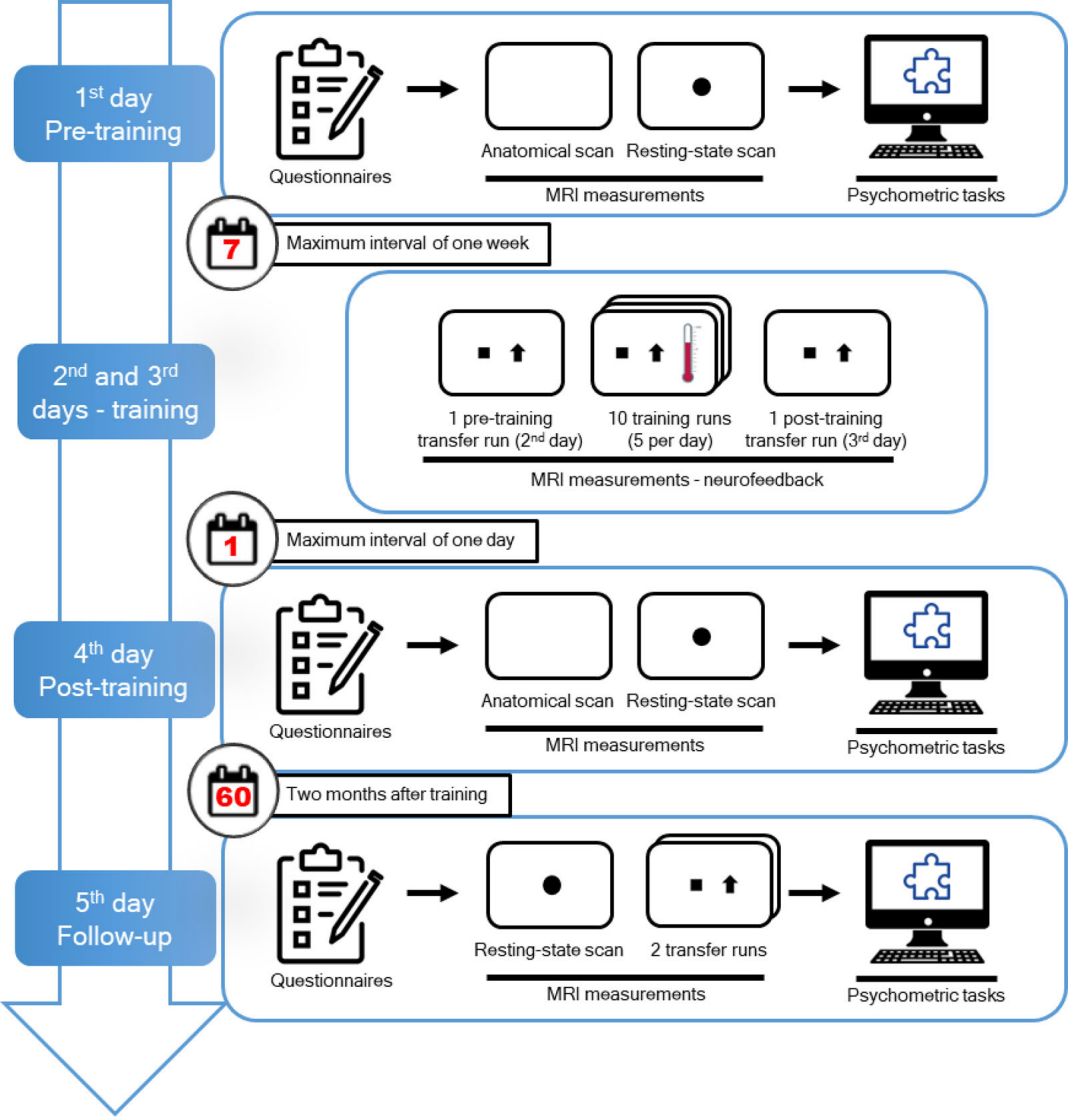
Timeline of the 5‐day neurofeedback experimental procedure. On the first, fourth, and fifth days of the experiment, participants underwent resting‐state functional MRI acquisitions and completed attention‐related questionnaires (Dundee Stress State Questionnaire and Cognitive Failures Questionnaire) as well as psychometric tasks on a computer (Continuous Performance Task, Switcher; Psychomotor Vigilance Test, Mental Rotation Task and Attentional Network Test). The neurofeedback training sessions occurred on the second and third days of the experiment and the participants performed self‐regulation without feedback during transfer runs directly before and directly after training. Additionally, participants also performed self‐regulation without feedback on two transfer runs on the fifth day. The five visits were conducted with a maximum of 1 week between the first and second days, 1 day between the third and fourth days, and 2 months between the third and fifth days. MRI, magnetic resonance imaging.

Each participant took part in a 5‐day longitudinal study (Figure 1) that involved fMRI‐neurofeedback training and pre/post‐training sessions for neural and behavioral assessment of neurofeedback training. The neurofeedback training consisted of 10 real‐time fMRI runs that took place on the second and third days of the experiment (Figure 1), split into five runs each day. Each neurofeedback training session lasted about 45 min. The interval between training days for each participant was a maximum of 7 days. Neurofeedback training runs consisted of five cycles of baseline, regulation, and intermittent feedback blocks, lasting 30, 40, and 4 s, respectively. To indicate the period of baseline, regulation, and feedback blocks, participants were presented with a black square, a black up‐arrow, and a graded blue‐to‐red thermometer on the center of a white screen, respectively. We acquired individual transfer runs—used to test learned self‐regulation in situations where feedback is not available—directly before and directly after training (second and third days of the experiment, respectively), as well as two transfer runs 2 months after the end of training (fifth day of the experiment). The transfer runs consisted of cycles of baseline and regulation, lasting 30 and 40 s, respectively, the same durations as in the training runs. Identical to the training runs, the baseline and regulation blocks were indicated in the transfer runs with a black square and a black up‐arrow, respectively, on a white screen. Transfer runs contained no feedback presentation blocks. On the first, fourth, and fifth days of the experiment, the participants filled attention‐related questionnaires (see Section 2.3) and underwent anatomical and resting‐state fMRI acquisitions. Attention tasks were performed outside the scanner. These measurements were made approximately at the same time of the day. The intervals between the first and second days, between the third and fourth days, and the third and fifth days were 7 days maximum, 1 day maximum, and 61 ± 3 days, respectively. Hence, the terms pre‐training, post‐training, and follow‐up correspond to measurements acquired on the first or second days, third or fourth days, and fifth day of the experiment, respectively. The pre‐ and post‐training transfer runs occurred on the same days as the neurofeedback training, which were the second and third days of the experiment, respectively. In contrast, the pre‐ and post‐training resting‐state runs as well as the psychometric tasks were performed 1 day before and 1 day after the neurofeedback training, which were the first and fourth days of the experiment, respectively (Figure 1). For the follow‐up session, the transfer runs, the resting‐state runs, and the psychometric task applications occurred on the same day (i.e., fifth day of the experiment).

#### 2.2.2 Instructions

Instructions for self‐regulation strategies during neurofeedback training were provided in written form outside the MR scanner room, prior to scanning. We instructed participants to relax and let their minds wander during baseline blocks and to engage in one of the suggested regulation strategies ((1) constantly reorienting the focus on different aspects of the arrow every 5–10 s; (2) focusing on the black up‐arrow and bringing attention back to it whenever detecting task‐unrelated thoughts; (3) staying in a state of high alertness) during regulation blocks. Participants were told that they could explore other regulation strategies and adopt the ones that worked best for them. Participants were also explicitly informed that, during baseline blocks, they should not plan regulation tasks. For the pre‐training transfer run, participants were asked to choose one of the suggested regulation strategies and employ it during this run. For the post‐training and follow‐up transfer runs, participants were asked to use the strategy that worked best throughout the neurofeedback training.

#### 2.2.3 MRI acquisition

All MRI data were acquired on a Philips Achieva 3T MRI scanner with a 32‐channel head coil in the MR center of the Psychiatric Hospital, University of Zürich, Switzerland. Functional images were acquired using a T2*‐weighted gradient‐echo‐planar imaging sequence with repetition time/echo time (TR/TE) = 2000/30 ms, flip angle = 80°, and field of view (FOV) = 240 × 240 mm^2^. Thirty‐seven slices were acquired in ascending order to cover the entire cerebrum (voxel size = 3 × 3 × 4 mm^3^, gap = 0.5 mm). SofTone mode was activated to reduce acoustic scanner noise. Anatomical T1‐weighted brain images were acquired using a 3D magnetization prepared gradient echo sequence, TR/TE = 7.2/3.4 ms, 170 slices, voxel size = 1 × 1 × 1 mm^3^, flip angle = 8°, FOV = 240 mm x 240 mm^2^, duration = 3.5 min. Resting‐state fMRI acquisitions comprised 200 scans (6 min 40 s) during which participants were asked to not move, to relax and breathe regularly, to look at a central black circle presented on a white screen for visual fixation, and not to think about anything in particular. Neurofeedback training and transfer acquisitions comprised 190 scans (6 min 20 s) and 180 scans (6 min), respectively. Before every functional acquisition, five dummy scans were performed to establish steady‐state magnetizations, as implemented in the Philips Achieva system, and not saved for real‐time processing or offline analyses. Visual stimuli were presented with MR‐compatible goggles (Resonance Technology Inc.).

#### 2.2.4 Definition of target functional networks involved in sustained attention

To improve sustained attention through neurofeedback training, we simultaneously promoted the activation of four representative regions of interest (ROIs) from the SAN and the deactivation of four representative ROIs from the DMN, areas positively and negatively associated with sustained attention performance. The SAN ROIs were defined using a mask of meta‐analytic clusters from a comprehensive study on sustained attention (Langner & Eickhoff, 2013; Table S1). The selected SAN ROIs were the anterior mid‐cingulate cortex (aMCC), the right inferior frontal junction (R IFJ), the right temporoparietal junction (R TPJ), and the right intraparietal sulcus (R IPS), chosen to represent multiple functional aspects of the ability of sustained attention. The aMCC is related to conflict processing, monitoring performance, and enhanced vigilance (Hinds et al., 2013; Langner & Eickhoff, 2013; Weissman et al., 2006); the R IFJ is related to stimuli discrimination and attention switching (Langner & Eickhoff, 2013); the R TPJ is associated with bottom‐up attention reorienting (Corbetta & Shulman, 2002; Weissman et al., 2006); and the R IPS is associated with top‐down attention reorienting (Corbetta & Shulman, 2002; Harris et al., 2000). The aMCC and R IFJ ROIs were spatially eroded from the original meta‐analytic clusters to increase their likelihood of being associated with sustained attention and to reduce possible differences in the difficulty of regulating the two networks involved in sustained attention. The selected DMN ROIs were the posterior cingulate cortex (PCC), medial prefrontal cortex (mPFC), and bilateral angular gyri (L Ang and R Ang). These regions are the most consistently reported DMN regions—the so‐called core regions of the DMN—and are robustly activated during self‐generated tasks (Andrews‐Hanna et al., 2014), in contrast to externally oriented attention tasks. To account for individual differences, the DMN ROIs were defined using the resting‐state acquisitions from each participant. More specifically, we first performed an independent component analysis (ICA) as implemented in Gift (mialab.mrn.org/software/gift) with a predefined number of 30 components. Next, using the Personode toolbox (Pamplona et al., 2020b; www.nitrc.org/projects/personode), we created 6‐mm‐radius spherical ROIs centered on probabilistic peaks that maximally represented each DMN regions for each individual (Table S1). The SAN and DMN target ROIs are depicted in Figure S1.

#### 2.2.5 Feedback estimation and presentation

On the first day, we acquired an anatomical image. Then, using SPM12 (www.fil.ion.ucl.ac.uk), we used the anatomical image to create an inverse deformation field file. We used this inverse deformation field to transform the ROIs, originally defined in the Montreal Neurological Institute (MNI) space, as well as the anatomical image, onto the subject space. Prior to the real‐time fMRI workflow in each training day (i.e., from the second day on), we also acquired one T2*‐weighted image, the so‐called “one‐volume” functional image. Using the subject‐space‐transformed anatomical image, the ROIs were coregistered to the day‐specific “one‐volume” functional image. “During training and transfer runs, functional images in Analyze format were transferred to the real‐time processing computer as soon as they were acquired and reconstructed in the MRI scanner computer via the Philips Direct Reconstructor Interface application. On this computer, spatial realignment (of subsequent real‐time functional images to the “one‐volume” functional) image, estimation of six movement parameters (translation and rotation), reslicing, and spatial smoothing with an isotropic Gaussian kernel with 5‐mm full width at half maximum (FWHM) were performed using OpenNFT (Koush et al., 2017) (Pamplona, Heldner et al., 2020)”. Spatial smoothing in real‐time fMRI processing was used to improve the correspondence between the SAN ROIs, based on a meta‐analytic definition, and the anatomical representation of the individual. Therefore, during the real‐time workflow, the voxel values from the real‐time realigned functional images masked with the ROI images coregistered to the subject space were acquired for further processing.

“Auto‐regressive correction of first order was performed online to reduce temporal autocorrelation caused by physiological noise (Lindquist, 2008), and an incremental general linear model (GLM) was used to remove residual motion and linear trends (Hinds et al., 2011). Spike detection and high‐frequency noise removal were performed through a modified Kalman filter (Koush et al., 2012) (Pamplona, Heldner et al., 2020)”.

For neurofeedback training runs, the signal averaged within each ROI was rescaled in real time (Koush et al., 2012; Pamplona, Heldner et al., 2020; Scharnowski et al., 2012). Next, the resulting signals were averaged within SAN and DMN separately. Finally, the difference between SAN and DMN signals (differential SAN minus DMN activity) was fed back intermittently to the participant as the thermometer level, right after regulation blocks. Participants were asked to raise the thermometer level as much as possible, which could be achieved either by SAN upregulation, DMN downregulation, or both. The thermometer level, comprised 10 negative (for DMN > SAN), zero, and 10 positive readings (for SAN > DMN), was proportional to the participant's performance in the current block. Feedback presentation was adaptive for each run based on performance in previous runs, that is, feedback was made more difficult if the task was relatively easy for the participant, and vice‐versa. At the end of each run, a monetary reward proportional to their performance in each run was shown to the participant (CHF 20.6 ± 5.4 in total per participant) and added to the final compensation to the participation.

### 2.3 Psychometric tasks and questionnaires

To evaluate mental strategies associated with neurofeedback training, we asked participants to report the used strategies immediately after each neurofeedback training run. In addition, at the end of training and transfer runs, participants rated their level of concentration on the previous run on a scale ranging from 1 (*very low*) to 10 (*very high*). Self‐reported concentration ratings from two participants were not collected due to technical issues with the communication system.

At the beginning of the first, fourth, and fifth days, participants also completed attention questionnaires (Cognitive Failures Questionnaire [CFQ]; Broadbent et al., 1982) and their current state of attentiveness and stress in real‐life situations (Dundee Stress State Questionnaire [DSSQ]; Helton, 2004). Technical failures in the acquisition led to incomplete data collection: inclusion of 14–15 participants in the pre‐training session, 6–7 participants in the post‐training session, and 12–15 participants in the follow‐up session (the number of participants varies depending on missing data specific to the sub‐score).

At the end of the first, fourth, and fifth days, participants performed five attention‐related tasks as implemented in the Psychology Experiment Building Language (PEBL) software (Langner et al., 2023; Mueller & Piper, 2014), outside the scanner (Figure 1). Attention tests were performed on a dedicated computer and in a separate experimental room with constant luminosity and noise (participants were asked to use earplugs). The selected tasks from PEBL were: (1) Continuous Performance Task (Conners et al., 2003; Ogg et al., 2008; Piper et al., 2016), a go/no‐go task designed to measure the sustained ability to either execute or withhold a speeded response; (2) Task‐Switching Performance (Switcher; Anderson et al., 2012), designed to evaluate the cognitive flexibility in reorienting attention to switching rules; (3) Psychomotor Vigilance Test (PVT; Dinges & Powell, 1985; Helton et al., 2007; Loh et al., 2004), designed to measure the level of alertness and its maintenance over time (sustained attention); (4) Mental Rotation Task (Berteau‐Pavy et al., 2011; Shepard & Metzler, 1971), designed to evaluate the visual imagery ability in transforming spatial characteristics of an image; (5) Attentional Network Test (ANT ; Fan et al., 2002), designed to provide measurements of different facets of attention: phasic alerting, endogenous spatial orientating, and conflict resolution. The tasks were presented always in the same order. To avoid fatigue, there were 5‐min breaks between the second and third tests and between the fourth and fifth tests.

### 2.4 Data analysis

Functional images from the transfer and resting‐state runs as well the anatomical images were preprocessed using SPM12 in MATLAB (The MathWorks). First, functional images were slice‐time‐corrected using the middle slice as a reference. Then, three translation and three rotation parameters of head motion were estimated, and the functional images were spatially realigned to a created mean functional image. Next, the anatomical image was coregistered to the mean functional image and then segmented into tissue probability masks for gray matter, white matter, and cerebrospinal fluid (CSF) compartments. During the segmentation process, a deformation field was created, which was used to normalize the anatomical and functional images to the standard MNI template. Finally, the normalized functional images from the transfer runs were spatially smoothed using a Gaussian kernel of 8 mm FWHM, and the normalized functional images from the resting‐state runs were smoothed with a kernel of 6‐mm FWHM. A larger kernel for activation analysis, compared to the connectivity analysis, was used because we expected large‐scale network differences in activation analyses and seed‐to‐voxel effects in smaller regions in connectivity analyses, despite the risk of sensitivity loss (but no risk of inflation of false positives; Alahmadi, 2021).

#### 2.4.1 Transfer run activity and regulation‐specific FC (regFC) analyses

##### First‐level analysis of transfer runs

We investigated differences in training‐induced neural activity changes across pre‐training, post‐training, and follow‐up transfer runs. For the first‐level analysis, we specified for each run a general linear model (GLM) with two regressors of interest representing regulation and baseline conditions and six covariates representing head motion. Regressors of interest were modeled as boxcar functions and convolved with the canonical hemodynamic response function implemented in SPM12. Next, beta values (regression weights) of regulation and baseline blocks for each participant and run were estimated voxel‐wise. Contrasts were created for the activation differences between regulation and baseline blocks for each participant and run.

##### Long‐term effects of regulation in trained networks

To examine the follow‐up effects in brain self‐regulation after neurofeedback training, we investigated whether the differential SAN‐DMN activity, as well as activations within the SAN and the DMN and their constituent ROIs, differed between follow‐up and the pre‐training transfer runs. First, contrast values (regulation vs. baseline) were extracted using MarsBaR (marsbar.sourceforge.net; Brett et al., 2002). Then, we averaged the contrasts from the four SAN and the four DMN ROIs to compute the SAN and DMN contrasts for each session, respectively, as well as the differential SAN‐DMN signal. The contrasts from the two follow‐up transfer runs were analyzed collapsing them together, as well as separately. We then compared the differential SAN‐DMN signal, as well as the contrasts for SAN and DMN and for their constituent ROIs separately, across follow‐up and pre‐training sessions using paired *t*‐tests using RStudio (www.rstudio.com). The normality of each run‐specific distribution was verified using Shapiro–Wilk tests. Statistical tests of the comparison of activity during follow‐up, compared to pre‐training sessions, were one‐tailed because we hypothesized more positive estimates for differential SAN‐DMN activity difference and for SAN activity, as well as more negative estimates for the DMN activity. We also estimated the effect sizes of the follow‐up minus pre‐training differences using Cohen's *d*.

##### Long‐term effects of regulation across the whole brain

First, individual contrast maps (regulation vs. baseline) for each session (i.e., pre‐training, post‐training, and follow‐up) were entered into a second‐level analysis in which subjects were treated as random effects. Then, voxel‐wise one‐sample *t*‐tests were performed to map the group activations and deactivations for each session. We also created statistical maps comparing post‐ versus pre‐training sessions and follow‐up versus pre‐training sessions. These statistical maps were obtained by entering individual contrast maps (post‐ minus pre‐training or follow‐up minus pre‐training) as random effects in one‐sample *t‐*tests (which is equivalent to paired *t*‐tests with partitioned errors (Henson, 2015)). The contrasts from the two follow‐up transfer runs were analyzed collapsing them together, as well as separately. All resulting group‐level maps were submitted to the threshold‐free cluster estimation (TFCE) approach (voxel‐level threshold of *p* < .001 uncorrected for multiple comparisons, 10,000 permutations). This approach provides high sensitivity for detecting both large and small clusters (Smith & Nichols, 2009) and is particularly suitable for small sample sizes. The thresholded group‐level maps were anatomically labeled using the bspmview toolbox (www.bobspunt.com/software/bspmview/; Spunt, 2016).

##### Changes in regFC across transfer runs

We applied a psychophysiological interaction (PPI) analysis to investigate changes in FC between target SAN/DMN ROIs and the whole brain, modulated by task blocks during transfer runs (McLaren et al., 2012; O'Reilly et al., 2012), using the toolbox CONN (version 19.c; Whitfield‐Gabrieli & Nieto‐Castanon, 2012). Seed‐based PPI maps were estimated across pre‐training, post‐training, and follow‐up sessions using a two‐level analysis. As seeds, we defined the four ROIs that comprised the SAN and the four DMN ROIs that were targeted during neurofeedback training. The SAN and DMN regions were masked with subject‐specific gray matter maps prior to their time‐course extraction. For the first‐level analysis, the interactions between the task blocks and the time courses of the targeted regions were defined as regressors of interest in separate GLMs for each seed and the betas were estimated. Regressors of no‐interest were defined as the six realignment parameters, their first‐level derivatives, and the five principal components from white matter and CSF time‐series (Behzadi et al., 2007). Additional denoising included bandpass filtering (0.008–0.09 Hz), despiking, and linear detrending. For the second‐level analysis, beta images of all participants were entered into Wilks’ lambda tests (a multivariate approach alternative to the repeated‐measures ANOVA, robust against the violation of the compound‐symmetry assumption). The group variance was then inferred across pre‐training, post‐training, and follow‐up sessions for each seed. Thresholded statistical *t*‐value maps were generated using the Gaussian random‐field theory (Worsley et al., 1996) with a cluster‐level threshold of *p* < .05, family‐wise error (FWE)‐corrected for multiple comparisons, and a voxel‐level inclusion threshold of *p* < .001. Post hoc analyses were performed to determine pairwise differences within the resulting PPI clusters across sessions using the library “emmeans” in RStudio with *p* < .05, Tukey‐corrected for multiple comparisons. Brain areas where regFC changes were found were anatomically labeled using xjView (www.alivelearn.net/xjview).

#### 2.4.2 Resting‐state FC analyses

##### Changes in seed‐based rsFC

We used rsFC to investigate changes in FC between target ROIs and the whole brain at rest due to neurofeedback training. This rsFC analysis was performed using the CONN toolbox. Seed‐based rsFC maps were estimated using two‐level analyses across pre‐training, post‐training, and follow‐up sessions. As seeds, we defined the four ROIs that comprised the SAN and the four DMN ROIs that were targeted during neurofeedback training, masked with subject‐specific gray matter maps prior to their time‐course extraction. For the first‐level analysis, the seed‐based time‐courses were defined as regressors and beta values were estimated voxel‐wise for each participant and region using GLMs. The regressors of no‐interest included the six realignment parameters and their first‐level derivatives, and the five principal components from white matter and CSF time‐series. Denoising included bandpass filtering, despiking, and linear detrending. For the second‐level analysis, beta images of all participants were entered into a Wilks’ lambda test, and the group variance was inferred across sessions. Thresholded statistical *t*‐value maps were generated using Gaussian random‐field theory with a cluster‐level threshold of *p* < .05, FWE‐corrected for multiple comparisons, and a voxel‐level inclusion threshold of *p* < .001. Post hoc analyses were performed to determine pairwise differences across sessions within the thresholded clusters. Brain areas where rsFC changes were found were anatomically labeled using xjView.

##### Changes within‐ and between‐network rsFC

We investigated training‐induced modulations in rsFC within and between canonical resting‐state networks over sessions. For this purpose, we estimated rsFC among canonical resting‐state networks, including DMN, DAN, FPCN, and SAL. We first calculated individual and group maximally independent spatial maps using ICA, as implemented in GIFT (trendscenter.org/software/gift/). For this, preprocessed resting‐state functional images were concatenated across sessions (pre‐training, post‐training, and follow‐up) for each participant and used for the ICA computation. We predefined the number of 25 spatial independent components to be obtained. Spatial IC maps were then classified into the canonical resting‐state networks of interest and their clusters (10 clusters for DAN, six clusters for DMN, 10 clusters for FPCN, and four clusters for SAL) were defined in an individualized way, as implemented in Personode (github.com/gustavopamplona/Personode; Pamplona, Vieira et al., 2020). For each participant and session, the average time‐courses from each cluster were extracted and denoised (as described in the paragraph above). Fisher‐transformed association matrices of FC were computed through correlations among every possible combination of clusters for each participant and session. Then, for each session, bootstrapping (using a customized code in MATLAB) was used to compute the distribution after one million permutations of the FC values across subjects within networks—that is, using FC values from combinations of clusters within the same network—and between networks—that is, using FC values from combinations of clusters from each possible pair of clusters from different networks. The same procedure was made for the association matrices of the differences in post‐ minus pre‐training sessions and follow‐up minus pre‐training sessions. Values from the upper triangular association matrices were included in the bootstrapping. Bootstrapping was used to compute the average and confidence intervals of FC for each session and for the differences between post‐ minus pre‐training and follow‐up minus pre‐training sessions. These differences were considered significant when the zero‐value was not within the confidence interval for a significance level of 0.05, corrected for multiple comparisons using the Bonferroni correction (two comparisons x [four within‐network + six between‐network computations], 20 in total).

##### Changes in rsFC within the SAN and DMN ROIs

We investigated modulations in rsFC within the SAN and DMN ROIs across pre‐training, post‐training, and follow‐up sessions using a graph theoretical approach. In graph theory applied to neuroimaging, the degree of FC is defined as the number of edges of an individual node for a given network and a given threshold (Rubinov & Sporns, 2010). Here, the degree of FC estimates to which extent a target network region is connected to the rest of the brain. We computed the degree of rsFC using the intrinsic connectivity distribution (ICD) approach (Scheinost et al., 2012), which does not require the choice of an arbitrary threshold. Specifically, for this analysis, slice‐time‐corrected and realigned resting‐state functional images were first normalized and resampled to a voxel size of 4 × 4 × 4 mm^3^, to reduce computational load in ICD computation, and smoothed using a kernel of 8 mm FWHM. The ICD was computed voxel‐wise and for each participant and session using a customized code as reported in Scheinost et al. (2012). To assess changes in the degree of SAN and DMN regions, we averaged the ICD voxel values within these regions for each participant and session. One‐way repeated‐measures ANOVAs were computed for each region, with session being defined as within‐subject factor. Post hoc analyses were performed to determine pairwise differences across sessions. *p‐*values were adjusted for multiple comparisons at the region level using the Tukey method. We estimated effect sizes for the main effect and the pairwise comparisons, that is, partial *η* ^2^ and Cohen's *d*, respectively.

#### 2.4.3 Analysis of behavioral effects

We investigated changes in sustained attention across sessions as measured by PVT. We previously reported that sustained attention improved right after neurofeedback training (Pamplona, Heldner et al., 2020). Specifically, participants improved in the first few minutes of the PVT task, but this improvement was no longer found in later trials of the PVT. We have showed in our previous study that PVT was the only task whose improvement could be related to neurofeedback training and not due to test–retest effects (Pamplona, Heldner et al., 2020; see also Langner et al., 2023). Therefore, we tested here whether this initial improvement in PVT persisted in follow‐up sessions. We did not analyze data from the other psychometric tasks applied because we did not observe short‐term neurofeedback‐induced changes in these tasks. We used a linear mixed effects model to account for the hierarchical structure (multiple measurements of response time for each subject), with the fixed effects Session and Trial and Subjects as a random effect; Trial being a continuous variable. Since we were interested in differences in reaction time (RT) over trials across sessions, we checked whether the two‐way interaction Session x Trial was significant. We then performed post hoc analyses to pairwise compare the RT across sessions at early and late trials separately. The post hoc analysis of Trial as a continuous variable was performed following the procedures described in (Cohen & Cohen, 1983; West et al., 1996); that is, early and late trials were defined as the average trial minus and plus one standard deviation, respectively. For linear mixed model and post hoc analyses, we used the libraries “lme4” and “emmeans” in RStudio (adjusted *p*‐values for multiple comparisons using the Tukey method), respectively. Effect sizes for post hoc analysis following linear mixed models were estimated with the library “emmeans.”

In addition, we investigated changes in self‐reported attention, namely, those DSSQ sub‐scores that were thought to be modulated between follow‐up and pre‐training sessions (i.e., motivation, self‐focused attention, concentration, control and confidence, task‐related interference). The other DSSQ scores are not specific to relevant attention measures and were not tested. Also, the CFQ scores were not tested here because they are assumed to be stabile over long periods (Broadbent et al., 1982). We used paired *t*‐tests and dependent two‐group Wilcoxon signed‐rank tests for parametric and nonparametric distributions, respectively, as assessed by Shapiro–Wilk tests. For each analysis, the *p*‐values were adjusted for multiple comparisons using the false discovery rate (FDR). Furthermore, we semantically compared and described the most reported strategies for both regulation and baseline blocks, as well as how many participants kept the same strategy in the follow‐up transfer runs, compared to the post‐training transfer runs. We also separated the participants into two groups, one that comprised participants that reported using the same strategies in both post‐training and follow‐up transfer runs and one that reported different strategies, and compared the betas of regulation performance between groups with a two‐sample *t*‐test. In addition, we compared the self‐rated concentration level between pre‐training and follow‐up transfer runs with a paired *t*‐test.

Finally, we performed an exploratory analysis in which we investigated associations of improved sustained attention with changes in FC between the DMN ROIs and the occipital gyrus. We first computed the absolute changes (i.e., the simple difference) of the average RT during the first half of the PVT for post‐ minus pre‐training sessions and for follow‐up minus pre‐training sessions. Only the first half of the PVT was considered here since we observed attentional improvement after neurofeedback training only during the first minutes of its application. We then computed the absolute changes of regFC estimates between the occipital gyrus and the PCC, the L Ang, and the R Ang, as well the absolute change of rsFC estimate between the occipital gyrus and the R Ang, for post‐ minus pre‐training sessions and for follow‐up minus pre‐training sessions. These regFC and rsFC estimates were selected because of the significant findings between DMN ROIs and the occipital gyrus (Figures 4 and 5). Finally, we computed the Spearman's correlation between PVT RT and FC estimates separately for absolute changes post‐ minus pre‐training sessions and follow‐up minus pre‐training sessions. The *p*‐values were adjusted using FDR for the multiple comparisons post‐ minus pre‐training sessions and follow‐up minus pre‐training sessions, separately.

#### 2.4.4 Summary of statistical methods

To assess the long‐term effects of regulation in trained networks, we compared ROI‐specific activity between follow‐up and pre‐training sessions using one‐tailed paired *t*‐tests, after testing for normality with Shapiro–Wilk tests, and calculated effect sizes using Cohen's *d*. To assess the long‐term effects of regulation across the whole brain, we determined session‐specific group activations and deactivations, as well as between‐session comparisons, using voxel‐level one‐sample and paired *t‐*tests and the TFCE approach with an inclusion threshold of *p* < .001 and 1 × 10^5^ permutations. We assessed changes in regFC (PPI estimates) and rsFC (FC estimates) across transfer runs for each seed‐specific maps using Wilks’ lambda tests and random‐field theory with a voxel‐level inclusion threshold of *p* < .001 and a cluster‐level FWE‐corrected threshold of *p* < .05; post hoc analyzed to determine pairwise differences in significant clusters with Tukey‐corrected *p* < .05. We assessed changes in within‐ and between‐network rsFC for four brain networks across sessions using bootstrapping with 1 × 10^6^ permutations to calculate mean and confidence intervals to a significance level of 0.05, with Bonferroni correction. To assess changes in rsFC within the SAN and DMN ROIs, we compared ROI‐specific ICD estimates across sessions using one‐way repeated‐measures ANOVAs with partial *η* ^2^ as a measure of effect size, post hoc analyzed to determine pairwise differences with Tukey‐corrected *p* < .05 and their effect sizes with Cohen's *d*. We assessed changes in sustained attention across sessions using a linear mixed effects model to check for a significant interaction Session x Trial, post hoc analyzed for early and late trials, and calculated Cohen's *d* as estimates of effect size. We assessed changes in self‐reported attention (DSSQ sub‐scores) and the self‐rated concentration level between pre‐training and follow‐up sessions using paired *t‐*tests and two‐group Wilcoxon signed‐rank tests for parametric and nonparametric distributions, respectively, with the *p*‐values adjusted using FDR. We compared regulation performance estimates between groups reporting the same and different strategies across sessions with a two‐sample *t*‐test. Finally, we assessed associations of improved sustained attention and changes in FC between the DMN ROIs and the occipital gyrus using the Spearman's correlation between PVT RT and FC estimates for absolute changes post‐ minus pre‐training and follow‐up minus pre‐training sessions, *p*‐values adjusted using FDR.

## 3 RESULTS

### 3.1 Long‐term effects of neurofeedback training during follow‐up transfer runs

#### 3.1.1 Long‐term effects in the trained networks during follow‐up transfer runs

**FIGURE 2:**
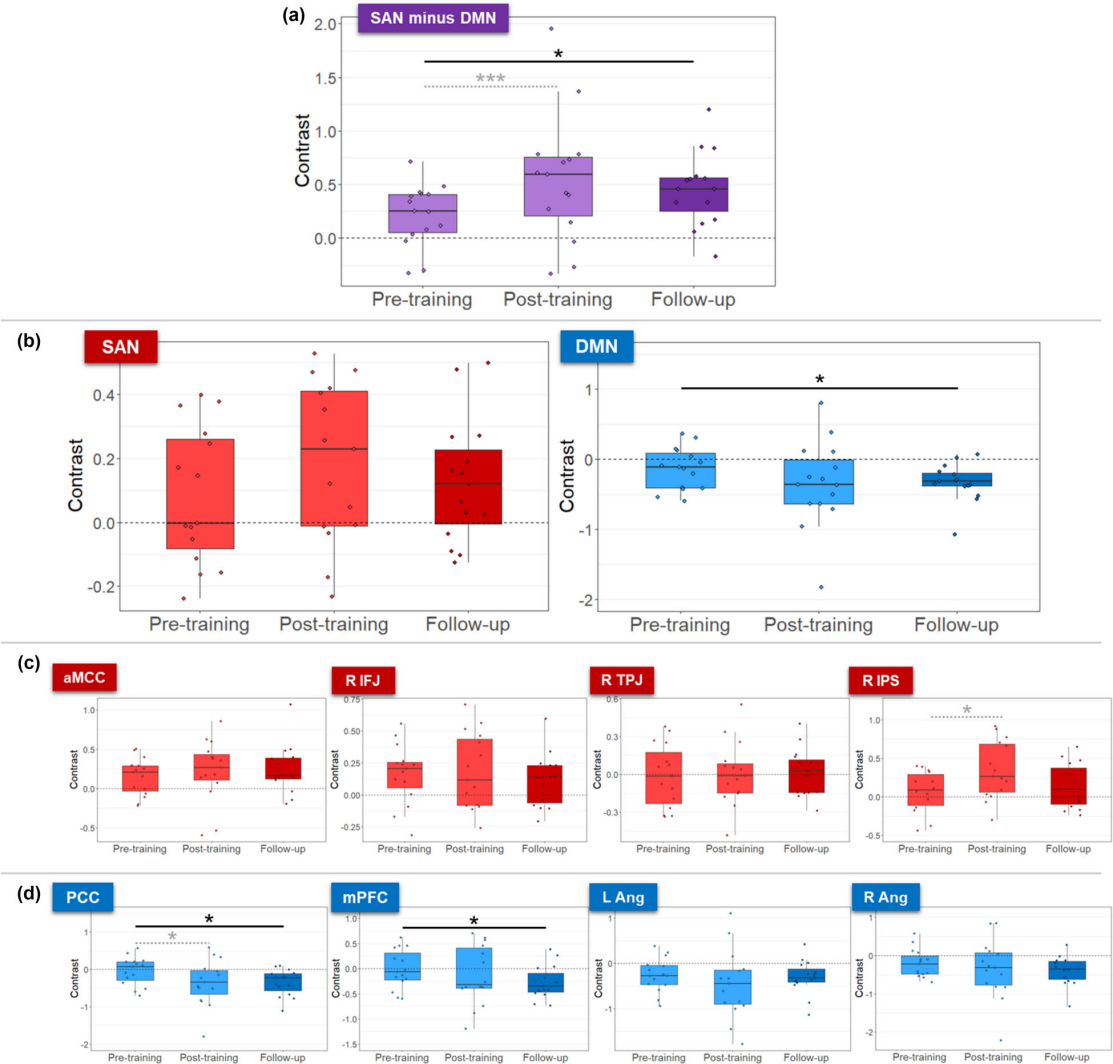
Learned self‐regulation of the differential SAN‐DMN activity was maintained during follow‐up transfer runs 2 months after neurofeedback training (a). Self‐regulation was mainly driven by down‐regulation of the DMN (b). No individual regions of interest (ROIs) from the SAN showed significant differences across sessions (c). The posterior cingulate cortex (PCC) and the medial prefrontal cortex (mPFC) as part of the DMN showed maintained down‐regulation during follow‐up runs (d). The graphs show the activation contrast between regulation and baseline blocks for pre‐training, post‐training, and the two follow‐up transfer runs. Purple and blue colors represent the differential SAN‐DMN activity and DMN regions, respectively. Light and dark colors represent pre‐/post‐training and follow‐up sessions, respectively. Gray dashed significance lines represent differences we have previously reported (Pamplona, Heldner et al., 2020). Asterisks indicate significant session differences (*** *p* < .001, * *p* < .05, uncorrected). aMCC, anterior midcingulate gyrus; DMN, default mode network; L Ang, left angular gyrus; R Ang, right angular gyrus; R IFJ, right inferior frontal junction; R IPS, right intraparietal sulcus; R TPJ, right temporoparietal junction; SAN, sustained attention network.

Previously, we found that participants learned to control the differential SAN‐DMN activity, mainly through down‐regulating the DMN (Pamplona, Heldner et al., 2020). Here, our new results show that learned self‐regulation of the differential SAN‐DMN activity was maintained during transfer runs without feedback 2 months after the neurofeedback training (paired *t*‐test between pre‐training and follow‐up runs: *t*(14) = 1.92, *d* = 0.51, *p* = .038 (Figure 2a). Also during follow‐up runs, self‐regulation was primarily driven by a persistent down‐regulation of the DMN (paired *t*‐test between pre‐training and follow‐up runs: *t*(14) = −1.80, *d* = −0.46, *p* = .047 (Figure 2b). Compared to pre‐training, self‐regulation of the SAN activity was neither different during post‐training runs nor during follow‐up runs (paired *t*‐test between pre‐training and follow‐up runs: *t*(14) = 0.59, *d* = 0.15, *p* = .28; Figure 2b).

When analyzing self‐regulation performance of each of the SAN and DMN ROIs separately, we observed that increased ability in down‐regulating the PCC was maintained during follow‐up runs (*t*(14) = 2.49, *d* = −0.64, *p* = .013; Figure 2c). Furthermore, the mPFC was down‐regulated during follow‐up (*t*(14) = −2.94, *d* = −0.50, *p* = .037; Figure 2c) but not during the post‐training session. No other regulation effects within SAN/DMN ROIs changed significantly across transfer runs (Figure 2c,d). Considering the follow‐up runs separately (i.e., without averaging the activation estimates across runs), we observed that the learned down‐regulation of the DMN and the mPFC was maintained only for the first follow‐up run (Figure S2), probably because of the lower number of subjects who performed the second follow‐up run (*N* = 11, compared to *N* = 15 in the first follow‐up run).

#### 3.1.2 Long‐term effects during follow‐up transfer runs across the whole brain

**FIGURE 3:**
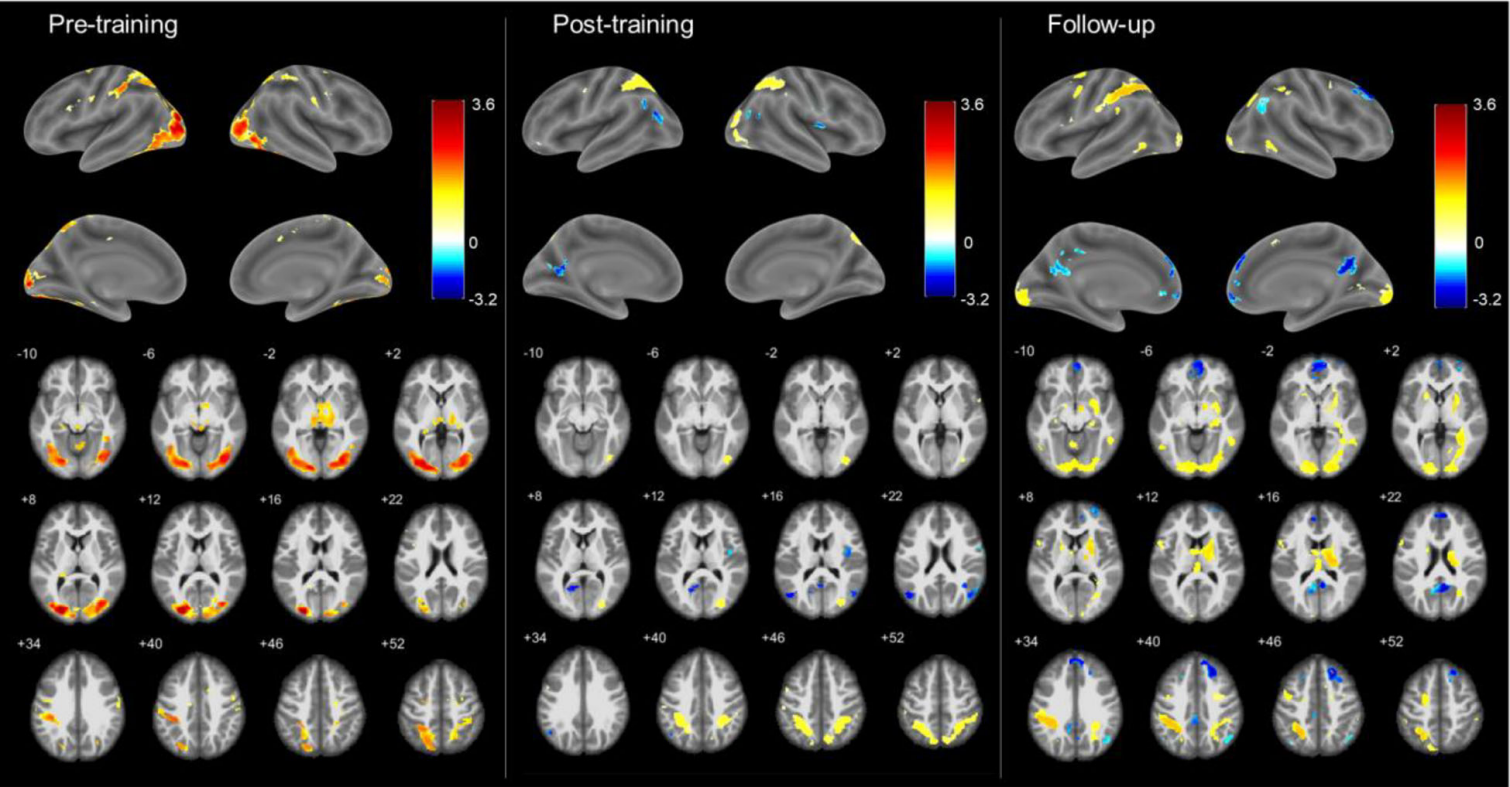
In whole‐brain analyses, down‐regulation of the default mode network during regulation compared to baseline was more strongly detected in follow‐up transfer sessions, compared to the pre‐ and post‐training transfer sessions. The dorsal attention network was activated in all sessions. Left, middle, and right columns show pre‐training, post‐training, and follow‐up transfer sessions, respectively. Hot and cold colors represent significant activation and deactivations during regulation, compared to baseline blocks, respectively, overlaid on surface‐rendered (top) and axial slices (bottom) from a brain template. T‐maps were generated by threshold‐free cluster enhancement, thresholded at *p* < .001 unc. for illustration.

Whole‐brain analyses showed significant deactivation (i.e., estimated betas at regulation < baseline) in the DMN during the follow‐up transfer session (i.e., averaged over the two follow‐up runs; Figure 3 and Table 1). Brain areas showing deactivation in the PCC and mPFC were larger in the follow‐up, compared to the post‐training session. While the right IPS was activated during all transfer runs and the bilateral angular gyri were deactivated during the post‐training run, all DMN ROIs were deactivated during follow‐up transfer runs. Activation in the DAN was detected in all transfer sessions (i.e., estimated betas at regulation > baseline). The thalamus was also activated in the follow‐up session. A complete list of activated and deactivated brain areas is reported in Table 1. The contrasts post‐ versus pre‐training and follow‐up versus pre‐training showed decreased activity in the left and right middle occipital gyrus, respectively, in post‐training and follow‐up, compared to the pre‐training sessions (Figure S3 and Table 1). Considering the follow‐up runs separately (i.e., without averaging the activation estimates across runs), we observed that the learned down‐regulation of the DMN was present in both follow‐up runs (Figure S4), despite lower activation effects, compared to Figure 3.

**TABLE 1:** Significant positive and negative activations during the pre‐training, post‐training, and follow‐up transfer sessions, as shown in Figure 3, including contrasts for the post‐ minus pre‐training and follow‐up minus pre‐training sessions as shown in Figure S3.

					Peak MNI coordinates		
Activation direction	Region label	Laterality	Extent	Peak *t*‐value	x	y	z
Pre‐training session							
Positive	Middle Occipital Gyrus, Inferior Temporal Gyrus	L	4629	3.56	−24	−90	6
	Inferior Occipital Gyrus, Fusiform Gyrus, Inferior Temporal Gyrus	R	3560	3.45	36	−76	−6
	Superior Frontal Gyrus	L	84	3.30	−20	−4	60
	Caudate Nucleus	R	596	3.30	18	−20	0
	Superior Parietal Lobule, Postcentral Gyrus	R	616	3.26	26	−40	48
	White matter	C	46	3.25	2	−36	−6
	Paracentral Lobule, Posterior‐Medial Frontal	C	114	3.24	−6	−38	70
	Cerebellum (VI)	L	51	3.23	−18	−60	−20
	Precentral Gyrus	L	71	3.22	‐42	‐4	32
	Hippocampus	L	50	3.19	−22	−38	6
	White matter	R	24	3.18	26	−28	2
	White matter	R	30	3.16	24	−14	50
	Inferior Frontal Gyrus (p. Triangularis)	L	100	3.14	−44	20	28
	Precentral Gyrus	R	105	3.13	58	−2	36
Post‐training session							
Positive	Middle Orbital Gyrus	R	152	3.06	26	42	−18
	Inferior Occipital Gyrus	R	220	3.06	40	−82	−8
	Middle Occipital Gyrus	R	205	3.06	28	−84	8
	Middle Occipital Gyrus, Precuneus	L	1604	3.06	−32	−44	36
	Inferior Parietal Lobule	R	1519	3.06	36	−44	38
	Middle Orbital Gyrus	L	54	2.88	−24	50	−14
	Precentral Gyrus	L	32	2.83	−48	−4	48
	Supramarginal Gyrus	L	55	2.77	−54	−30	48
Negative	Middle Temporal Gyrus	L	271	3.06	−46	−74	20
	Lingual Gyrus	L	144	3.04	−12	−58	8
	Middle Temporal Gyrus	R	233	3.01	48	−72	18
	Rolandic Operculum	R	90	2.98	42	−8	18
	Rolandic Operculum	R	24	2.77	62	0	20
Follow‐up							
Positive	Thalamus, Putamen	R	1526	3.22	16	−14	14
	Supramarginal Gyrus, Middle Occipital Gyrus	L	1302	3.22	−30	−44	32
	Fusiform Gyrus, Lingual Gyrus	R	4028	3.18	30	−52	2
	Superior Frontal Gyrus	L	327	3.16	−24	−12	52
	Superior Parietal Lobule	L	137	3.15	−30	−62	64
	Inferior Temporal Gyrus	L	50	3.13	−50	−64	−6
	Precentral Gyrus	L	97	3.11	−44	−4	46
	Supramarginal Gyrus	R	665	3.11	24	−54	40
	White matter	C	168	3.11	0	−22	16
	Putamen	L	106	3.09	−22	0	8
	White matter	L	28	3.09	−28	−66	26
	Inferior Temporal Gyrus	R	147	3.08	52	−52	−6
	Caudate	L	27	3.07	−16	0	18
	Superior Parietal Lobule, Precuneus	R	120	3.05	32	−60	62
	Precuneus	C	119	3.05	−8	−80	52
	Inferior Frontal Gyrus (p. Opercularis)	L	127	3.05	−54	10	12
	Cingulate Gyrus	R	24	3.04	16	−4	48
	White matter	R	93	3.00	28	2	40
	Postcentral Gyrus	L	34	2.99	−50	−38	56
Negative	Superior Frontal Gyrus, Superior Medial Gyrus	R	1032	3.22	20	34	46
	Precuneus	C	563	3.15	6	−58	22
	Middle Orbital Gyrus	C	443	3.06	6	60	‐6
	Posterior Cingulate Cortex (PCC)	C	152	2.98	2	−42	36
	Middle Frontal Gyrus	R	140	2.93	34	60	−2
	Middle Frontal Gyrus	L	30	2.87	−24	28	44
	Angular Gyrus	R	244	2.80	44	−66	34
	Angular Gyrus	L	23	2.53	−46	−68	42
Post‐ minus pre‐training							
Negative	Middle Occipital Gyrus	L	75	3.06	−34	−86	8
Follow‐up minus pre‐training							
Negative	Middle Occipital Gyrus	R	53	2.87	40	−78	8
	Inferior Occipital Gyrus	R	29	2.60	44	−70	−6

*Note*: Coordinates represent local maxima peak. Only clusters with more than 20 voxels are shown.

Abbreviations: L/R/C, left/right/center, MNI, Montreal Neurological Institute.

#### 3.1.3 regFC changes across transfer runs

**FIGURE 4:**
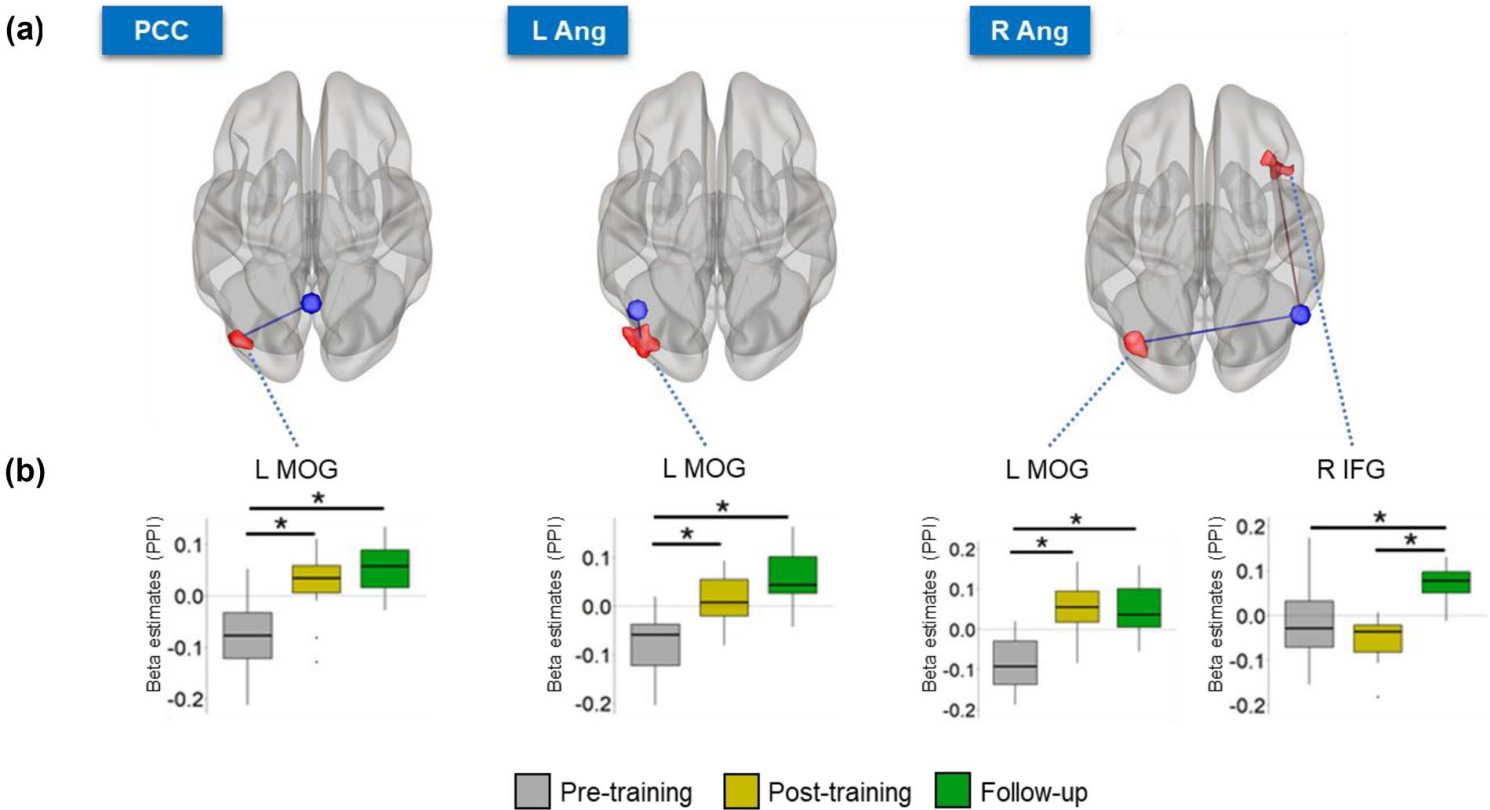
(a) Regulation‐specific FC (regFC) analysis showed increased FC between DMN regions (PCC, L Ang, R Ang) and the left middle occipital gyrus during post‐training and follow‐up transfer runs, compared to pre‐training runs. Blue and red regions represent DMN ROIs and significant seed‐to‐voxel FC regions, respectively, projected onto glass brains. (b) Boxplots represent the individual betas estimated for the PPI regressor of the DMN ROIs for each session; gray, yellow, and green represent pre‐training, post‐training, and follow‐up transfer sessions, respectively. Asterisks indicate significant differences corrected for multiple comparisons using the Tukey method (*p* < .05). Ang, angular gyrus; DMN, default mode network; FC, functional connectivity; IFJ, inferior frontal junction; L/R, left/right; MOG, middle occipital gyrus; PCC, posterior cingulate cortex; PPI, psychophysiological interaction.

Significant regFC changes between pre‐training, post‐training, and follow‐up transfer runs were found mainly between the DMN regions PCC, L Ang, R Ang and the left middle occipital gyrus (Figure 4). RegFC changes were also found between the SAN regions and the right angular gyrus, left hippocampus, and postcentral gyrus (Figure S5). No significant clusters of regFC were found having the mPFC as a seed and the statistical thresholds used. Summary group results of brain areas with significant regFC changes are shown in Table 2.

**TABLE 2:** Changes in regulation‐specific (regFC) and resting‐state (rsFC) functional connectivity between SAN/DMN and other brain areas across sessions, as illustrated in Figures S5, 4, and 5.

regFC												
	Connecting brain area			Follow‐up minus pre‐training			Post‐training minus pre‐training			Follow‐up minus post‐training		
Seed	Label	MNI peak coordinates (mm)	Extent (voxels)	*t*(28)	Cohen's *d*	*p*‐value	*t*(28)	Cohen's *d*	*p*‐value	*t*(28)	Cohen's *d*	*p*‐value
aMCC (SAN)	Right angular gyrus	(34, −66, 44)	120	+5.38	+2.16	<.0001	–	–	–	+3.97	+1.29	.0013
R IFJ (SAN)	Left postcentral gyrus	(−44, −30, 54)	142	−4.13	−1.41	.0008	–	–	–	‐5.73	‐2.29	<.0001
R TPJ (SAN)	Left hippocampus	(−18, −8, −20)	133	+4.86	+2.00	.0001	–	–	–	+4.66	+1.68	.0002
R IPS (SAN)	Left postcentral gyrus	(−44, −16, 48)	125	−3.16	−1.31	.010	+2.50	+1.00	0.05	‐5.66	−2.46	.0001
PCC (DMN)	Left middle occipital gyrus	(−42, −82, 12)	121	+4.20	+1.52	.0007	+5.44	+2.14	<0.0001	–	–	–
L Ang (DMN)	Left middle occipital gyrus	(−46, −82, 8)	165	+5.83	+2.09	<.0001	+3.97	+1.53	0.0013	–	–	–
R Ang (DMN)	Left middle occipital gyrus	(−40, −76, 10)	113	+4.79	+2.09	<.0001	+4.79	+2.08	<0.0001	–	–	–
	Right inferior frontal gyrus	(36, 24, −16)	136	+4.11	+1.45	.0009	–	–	–	+5.76	+2.69	<.0001
rsFC												
R Ang (DMN)	Left superior occipital gyrus	(−6, −94, 18)	87	–	–	–	+5.35	+1.23	<0.0001	−3.67	−0.87	.0028

*Note*: Results from post hoc analyses of the pairwise differences (follow‐up minus pre‐training, post‐training minus pre‐training, and follow‐up minus post‐training). Blank cells indicate non‐significant contrasts. aMCC, anterior midcingulate gyrus; DMN, default mode network; L Ang, left angular gyrus; MNI, Montreal Neurological Institute; R Ang, right angular gyrus; R IFJ, right inferior frontal junction; R IPS, right intraparietal sulcus; R TPJ, right temporoparietal junction; SAN, sustained attention network.

### 3.2 Changes in seed‐based rsFC

**FIGURE 5:**
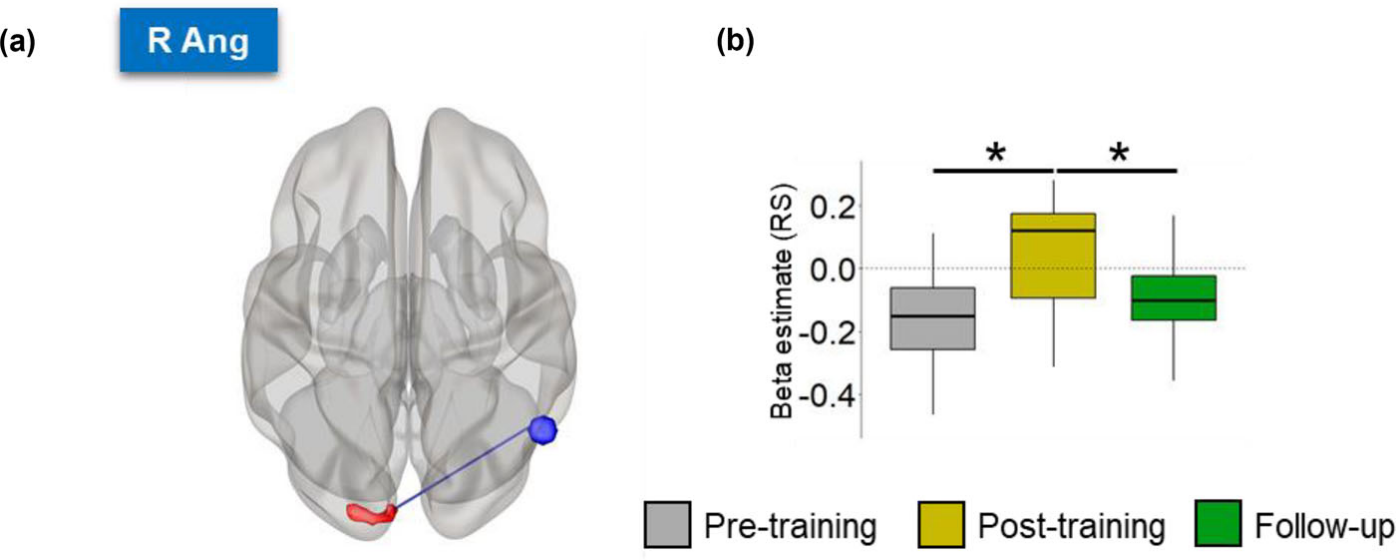
(a) Resting‐state functional connectivity (rsFC) between the right angular gyrus (R Ang) and the middle occipital gyrus increased during post‐training, compared to pre‐training runs, but returned to pre‐training levels during the follow‐up runs. Blue and red brain areas represent DMN regions and significant seed‐to‐voxel rsFC regions, respectively, projected onto a glass brain. (b) Boxplots represent the individual betas estimated for the regressor constructed with the average time course within the R Ang; gray, yellow, and green represent pre‐training, post‐training, and follow‐up sessions, respectively. The dashed black lines in the boxplots represent the zero level. Asterisks indicate significant differences corrected for multiple comparisons using the Tukey method (*p* < .05). DMN, default mode network; RS, resting state.

Significant seed‐based rsFC changes across pre‐training, post‐training, and follow‐up resting‐state sessions were found between the right angular gyrus and the superior occipital gyrus (Figure 5). No significant clusters of rsFC were found having the SAN ROIs, as well as the PCC, mPFC, and L Ang, as seeds and the statistical thresholds used. Summary group results of the regions with significant rsFC changes are shown in Table 2.

We defined individualized resting‐state large‐scale networks (DAN, DMN, FPCN, and SAL) for each participant and compared the FC within and between networks across sessions. We observed the expected positive correlation within networks, between DAN and FPCN, and between DMN and SAL, as well as the negative correlation between DAN and DMN (Table S2). However, we observed no evidence that the neurofeedback modulated either the within‐ or the between‐connectivity in the short or long term.

**FIGURE 6:**
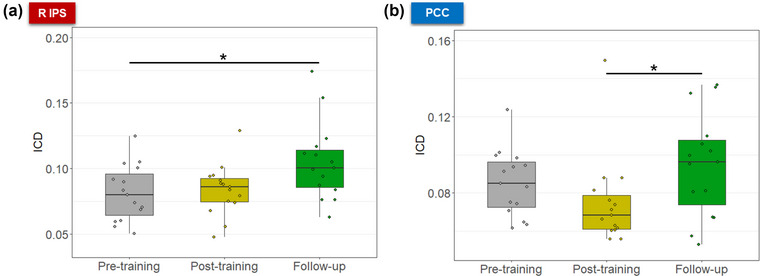
Higher degree of resting‐state FC was observed in the follow‐up session in (a) the right IPS (compared to pre‐training), and (b) the PCC (compared to post‐training). Asterisks indicate significant differences in post hoc analyses, corrected for multiple comparisons using the Tukey method (*p* < .05). ICD, intrinsic connectivity distribution; PCC, posterior cingulate cortex; R IPS, right intraparietal sulcus.

Graph‐theoretical analysis revealed that the degree of rsFC changed over the course of pre‐training, post‐training, and follow‐up resting‐state sessions in two of the trained regions: right IPS (*F*(2,28) = 4.40, *η* ^2^ = 0.24, *p* = .022) and PCC (*F*(2,28) = 3.86, *η* ^2^ = 0.22, *p* = .03; Figure 6). Post hoc analyses showed that the degree of rsFC increased in the right IPS from pre‐training to follow‐up (mean_pre‐training_ = 0.08 ± 0.02, mean_follow‐up_ = 0.10 ± 0.03, *t*(28) = 2.41, *d* = 0.93, *p* = .03) and in the PCC from post‐training to follow‐up (mean_post‐training_ = 0.07 ± 0.02, mean_follow‐up_ = 0.09 ± 0.02, *t*(28) = 2.78, *d* = 0.79, *p* = .03). No other significant differences in the degree of rsFC were found for the other target ROIs.

### 3.3 Behavioral effects cease to exist

**FIGURE 7:**
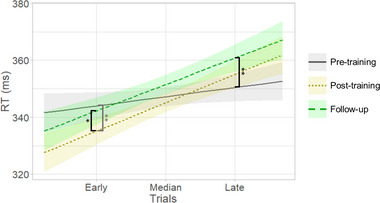
Differences between psychomotor vigilant test reaction time (RT) in pre‐training, post‐training, and follow‐up sessions indicate that improved sustained attention after neurofeedback training was no longer evident 2 months later. Also, during follow‐up, performance during late trials was worse, compared to pre‐training. Gray, yellow, and green colors represent measurements at pre‐training, post‐training, and follow‐up sessions, respectively. Asterisks indicate significant differences in post hoc analyses, corrected for multiple comparisons using the Tukey correction (** *p* < .01, * *p* < .05). The gray significance line represents a difference that we have previously reported (Pamplona, Heldner et al., 2020).

We previously found that neurofeedback training led to shorter RTs in early trials of the PVT (*d* = 0.15), indicating improved sustained attention in the first minutes of the task following neurofeedback training (Pamplona, Heldner et al., 2020). However, the training‐induced improved sustained attention in early trials of the PVT was not maintained in follow‐up tests 2 months after the training (Figure 7). Specifically, there was a significant interaction between the factors Day and Trial (*F*(1,5091) = 6.58, *p* = .0014). According to the procedure described for post hoc analysis following linear mixed models with continuous variables (Cohen & Cohen, 1983; West et al., 1996), RT for the PVT during early trials in the follow‐up was longer than in the post‐training session (*t*(5091) = 2.40, *d* = 0.12, *p* = .04; follow‐up: mean = 343 ms, confidence interval (CI) = [318, 368] ms; post‐training: mean = 336 ms, CI = [311, 361] ms) and was not different from the pre‐training session (*t*(5091) = 0.66, *d* = 0.03, *p* = .8; pre‐training: mean = 344 ms, CI = [320, 369] ms). In addition, the PVT RT during late trials was longer in the follow‐up, compared to the pre‐training session (*t*(5091) = 3.60, *d* = 0.18, *p* = .0010; follow‐up: mean = 361 ms, CI = [336, 386] ms; pre‐training: mean = 351 ms, CI = [326, 376] ms), and was not different from the post‐training session (*t*(5091) = 2.07, *d* = 0.10, *p* = .10; mean = 356 ms, CI = [331, 381] ms).

Attentional/motivational states, measured by the DSSQ, during follow‐up transfer runs were not different from pre‐training sessions (all FDR‐corr. *p*s > .05). A list of strategies used for regulation and baseline blocks during pre‐training, post‐training, and follow‐up transfer sessions is shown in Table S3. The most reported strategies for regulation blocks were keeping the attentional focus on the geometry of the up‐arrow (*N* = 6), thoughts related with past memories or future projection (2), and performing mental math (2). The most reported strategies for baseline blocks were trying to think about nothing in particular (4), mind‐wandering (3), and mental imagery of sports (2). Eight of the participants reported the same strategies (for both blocks) that they reported for transfer runs right after the end of the training. Considering strategies adopted in the follow‐up and the post‐training run, self‐regulation performance was not different between participants that used the same reported strategies and participants that used different ones (Welch two sample *t*‐test: *T*(7.4) = 0.07, *p* = .9). There were no differences between self‐rated concentration reported after pre‐training and follow‐up transfer runs (paired *t*‐test: *T*(12) = 0.97, *p* = .4).

**FIGURE 8:**
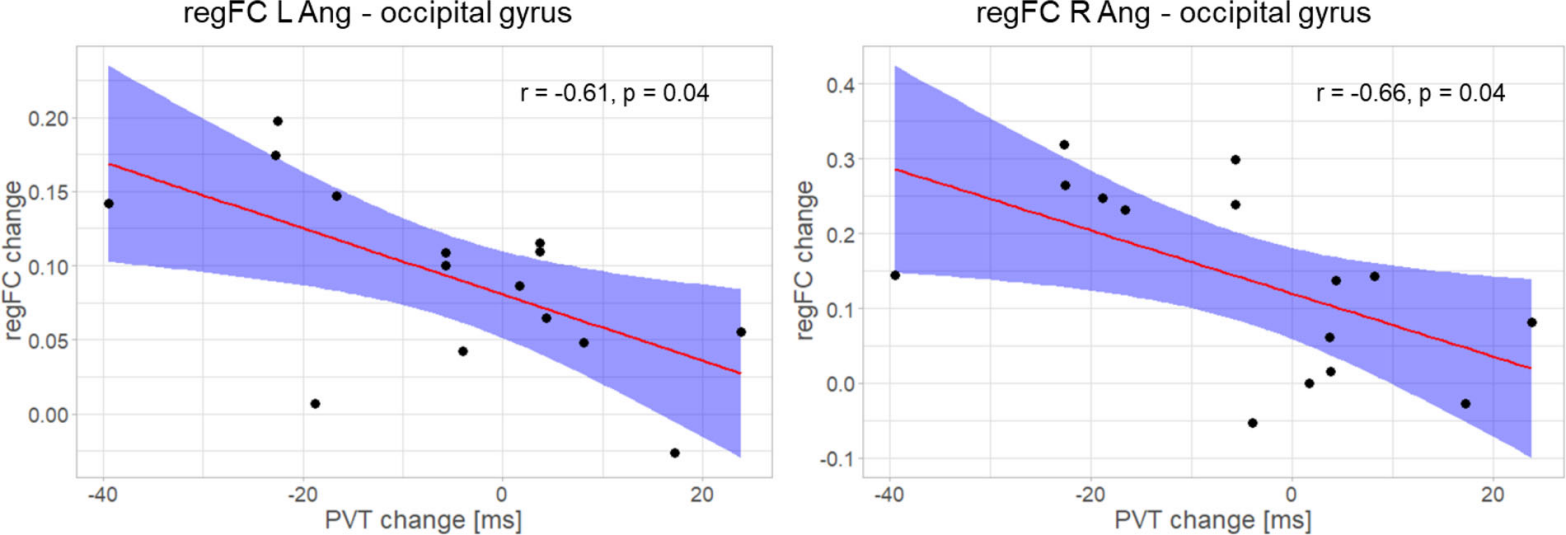
The change in the regFC between the bilateral angular gyri, brain areas that are part of the DMN, and the occipital gyrus was correlated with the RT change for the first half of the PVT across individuals, considering the difference post‐ minus pre‐training sessions. DMN, default mode network; L/R Ang, left/right angular gyrus; PVT, Psychomotor Vigilance Test.

In an exploratory analysis, we computed the correlation between behavioral changes (RT in the first half of the PVT) and connectivity only for the targeted DMN ROIs that showed significant results, that is, the regFC between the occipital gyrus and the PCC, the L Ang, and the R Ang (Figure 4) and the rsFC between the occipital gyrus and the R Ang (Figure 5). We found that the absolute change in the regFC between the L Ang/R Ang and the occipital gyrus was correlated with the absolute change in the RT in the first half of the PVT across individuals and considering the difference post‐ minus pre‐training runs (L Ang: *r* = −0.61, adj. *p* = .04; R Ang: *r* = −0.66, adj. *p* = .04, Figure 8). The significant negative correlation indicates that the degree of regFC increase during the post‐training run is associated with the degree of reduction in the RT. The change in the RT in the first half of the PVT was neither correlated with the post‐ minus pre‐training change in the regFC between the PCC and the occipital gyrus (*r* = −0.23, *p* = .5), nor with the rsFC between the R Ang and the occipital gyrus for the same period (*r* = −0.12, *p* = .7). Correlations considering follow‐up minus pre‐training changes were not significant (all *p*s > .05).

## 4 DISCUSSION

In this study, we investigated the maintenance of brain and behavioral changes associated with fMRI neurofeedback training on functional networks involved in sustained attention during transfer and resting‐state runs conducted before, immediately or 1 day after, and 2 months after the training. We found evidence for the maintenance of learned self‐regulation and lasting, plastic brain changes. Specifically, we found that after 2 months, participants were still able to up‐regulate the differential SAN‐DMN activity and that successful self‐regulation was driven mainly by down‐regulating the DMN. Also, the training‐induced increase in functional coupling between DMN and occipital cortex during transfer runs was maintained. Finally, the degree of FC during resting‐state runs increased in those brain regions that were successfully trained with neurofeedback. On the other hand, the increase in functional coupling between DMN and occipital cortex during resting‐state runs after training returned to baseline level during follow‐up runs. Behaviorally, the improved sustained attention right after neurofeedback training also returned to baseline level 2 months later.

### 4.1 Lasting neurofeedback effects on the differential SAN‐DMN activity

The ability to self‐regulate differential SAN‐DMN activity, acquired through neurofeedback training, was still present 2 months after training. This is in line with previous findings showing that brain self‐regulation learned through neurofeedback training is maintained for months (Amano et al., 2016; Robineau et al., 2017). Whereas these previous studies trained for three sessions, neurofeedback training in our study was limited to two sessions of 45 min each, showing that relatively short neurofeedback training allows participants to learn lasting self‐regulation skills (i.e., at least 2 months).

Learned self‐regulation of the differential feedback signal was primarily driven by down‐regulation of the DMN, which was observed right after training and during follow‐up 2 months later (Figures 2 and 3). Interestingly, DMN down‐regulation during transfer runs was even more pronounced during follow‐up, compared to right after training. For example, down‐regulation of the mPFC and right angular gyrus was observed only during follow‐up after 2 months but not directly after neurofeedback training (Figures 2d and 3). Akin to improvements following behavioral interventions, it might be that after training participants continue practicing self‐regulation in everyday life, thus further improving (Rance et al., 2018). Since activation in the DMN is associated with internally oriented attention (Bonnelle et al., 2011; Gusnard et al., 2001; Hinds et al., 2013; Mason et al., 2007), the improvement in DMN down‐regulation over time might reflect a reduced propensity for mind‐wandering in favor of a greater externally oriented attention. Therefore, learning and maintenance of self‐regulation of large‐scale networks might have implications in the ability of censoring spontaneous task‐irrelevant thoughts. However, these remain speculations as we currently do not have data on practice outside the experiment and on reduced mind‐wandering.

Apart from DMN down‐regulation, the only SAN ROI that was up‐regulated in the post‐training session was the right IPS. However, this was not maintained in the follow‐up session (Figure 2c). The IPS is part of the DAN, which was active during all transfer runs (Figure 3). DAN activity is related to the preparation and execution of top‐down or goal‐directed attention (Fox et al., 2005), and the IPS, specifically, is associated with reorienting top‐down attention (Corbetta & Shulman, 2002). At least 40% of the participants used a strategy that involves reorienting top‐down attention (“constantly reorienting the attentional focus on the geometry of the up‐arrow”; Table S3, thus likely resulting in activation of the DAN; Figure 3). The increased ability to activate IPS during the post‐training session, compared to the pre‐training session, might indicate improved engagement of neural resources mediating top‐down attention. However, the follow‐up session showed that the lasting effects were unrelated to the SAN but primarily driven by lasting increased DMN down‐regulation. Furthermore, although no differences across sessions were observed for activation in the aMCC, this region was stably engaged across sessions (Figure 2). The stable engagement of the aMCC in neurofeedback training has previously been suggested to be related to the process of learning a skill (Auer et al., 2018). Therefore, such an activation may reflect the aMCC as a mediator in the skill‐learning aspect of neurofeedback. The lack of lasting increased SAN up‐regulation might have been a consequence of having trained healthy participants with intact top‐down attention. It remains to be tested if our neurofeedback training in patients with attention deficits would lead to lasting SAN (and DMN) changes.

When comparing post‐training and follow‐up transfer runs to pre‐training transfer runs, we found reduced activity in the occipital gyri (Figures 3 and S3). Hence, for participants who focused their attention on visual features of the feedback display during training (Table S3), reduced occipital activity might indicate habituation to re‐occurring visual stimuli (Weigelt et al., 2008). Alternatively, for participants who focused on internally oriented attention (e.g., see participants #2 and #8 in Table S3), reduced occipital gyrus activity might also indicate reduced processing of visual information as a function of training sustained, internal‐focused attention (Benedek et al., 2016).

Finally, we observed increased thalamus activity during follow‐up transfer runs (Figure 3). Thalamic activity is related to general arousal maintaining alertness (Sarter et al., 2001). Thalamic activity during vigilant attention decreases over time but returns when new conditions are presented, playing a role in compensatory attentional effort (Langner & Eickhoff, 2013). Since we observed thalamic activity during self‐regulation a long time after the end of training, it might be that the thalamus activity was associated with arousal related to compensating for a less automatic state of sustained attention, compared to directly after training.

### 4.2 Lasting neurofeedback effects on regFC versus transient resting‐state FC and behavioral changes

Our neurofeedback training induced both short‐ and long‐term changes in regFC and rsFC (Table 2). Previous studies have reported lasting changes in FC due to neurofeedback training in patients (Scheinost et al., 2013; Yuan et al., 2014) and healthy participants (Megumi et al., 2015). As argued by Rance and colleagues, changes in FC may be continuously reinforced after neurofeedback training (Rance et al., 2018) over the course of days (Harmelech et al., 2013), weeks (Yuan et al., 2014), or months (Megumi et al., 2015). Our findings further support claims that neurofeedback can induce FC changes that are maintained for several months.

The most consistent FC changes that we observed were related to increased FC between the DMN and the occipital cortex (Figures 4 and 5). More specifically, we observed that the regFC between DMN and occipital cortex increased during post‐training and follow‐up runs, compared to pre‐training runs. A previous PPI study showed that the connectivity between DMN and the occipital cortex increases when the FPCN is engaged (Karten et al., 2013). It was suggested that the increase in the DMN–occipital cortex connectivity reveals a top‐down mechanism suppressing the bottom‐up visual stream (Karten et al., 2013) and protecting internal attentive processes from potentially distracting sensory stimulation (Benedek et al., 2016). In addition, the suppression of externally and internally distracting information, that is, generated in the visual cortex and the DMN, respectively, is closely linked to each other and predictive of task performance (Anticevic et al., 2012). Thus, the engagement of the FPCN during transfer runs, observed in all sessions (Figure 3), may also have increased connectivity between DMN and the occipital cortex. In our study, the SAN has components of the FPCN, specifically the aMCC and the right TPJ. However, we observed no differences in the engagement of FPCN across sessions (Figure 2). Therefore, the modulation of connectivity between DMN and the occipital cortex based on the engagement of the FPCN after neurofeedback training is only partially explained, and other factors should also be considered. For example, only a subdivision of the FPCN has previously been shown to be connected to the DMN (Dixon et al., 2018) and the engagement‐dependent modulation of the connectivity between the DMN and occipital cortex may require a more detailed analysis to be verified, which was beyond the scope of our study. Future studies may shed further light on this point. Furthermore, we observed that greater regFC between DMN and occipital cortex were associated with faster response time (Figure 8) when comparing post‐training with pre‐training sessions. Such an association might indicate that participants learned to simultaneously suppress distracting externally and internally information and that this ability was also employed during the sustained attention task. The fact that the behavioral effects were present during a task that was conducted 1 day after neurofeedback training suggests that these training effects might be lasting. However, these associations were not observed when comparing follow‐up and pre‐training sessions. Thus, while the improved regulation‐related FC was maintained in the long term, the improved attentional performance and the increased connectivity supporting an attentional state were not. This dissociation indicates that learned brain self‐regulation can be applied voluntarily but does not necessarily translate to situations without voluntary attention regulation in the long term.

Interestingly, we also observed FC changes in resting‐state runs following the end of training. More specifically, the rsFC between DMN and occipital cortex increased 1 day after the end of training but was not different from pre‐training runs 2 months after the end of training (Figure 5). Akin to the behavioral effects that did not last, also the rsFC changes that indicated plastic changes in the functional coupling between DMN and the occipital cortex were not maintained even though participants were still able to regulate and regFC changes persisted (Figure 4). The observation that changes in the regFC between DMN and the occipital cortex were maintained in the long term, while rsFC changes were not, is unlikely to be artifactual. First, because these differences in short‐ and long‐term effects are consistent with the behavioral findings: Increased regFC in the long term reflects the improved suppression of external distracting information toward a higher attentional state during the learned task‐related modulation, whereas measurements in which task‐related modulation is not overtly requested (i.e., rsFC and attention tests) did not show sustained long‐term improvement. Second, resting state measurements are expected to lead to less pronounced differences, compared to the regulation periods: While rsFC relates to plasticity and training‐specific induced changes, regFC relates to a newly acquired ability, which may be a more salient effect to be measured. Third, lasting changes in the degree of FC in successfully trained ROIs during resting‐state runs were observed (Figure 6): Because during resting‐state runs no active self‐regulation was required, such FC changes likely represent plastic brain changes that are unrelated to concurrent mental strategies activations. As the change in the degree of rsFC was specific to regions that were successfully trained with neurofeedback (Figure 2c,d), probably supporting the acquired ability to regulate brain activity. Previous studies have found lasting resting‐state changes following neurofeedback training (Megumi et al., 2015). Some recent studies have even reported brain structural changes associated with neurofeedback training (Papoutsi et al., 2018; Sampaio‐Baptista et al., 2021), showing the potential of neurofeedback to produce lasting effects on brain structure and processing.

It is noteworthy that the proposed neurofeedback training did not change the coupling within and between large‐scale networks, neither in the short nor in the long term (Table S2). Therefore, together with our other findings, we conclude that the proposed neurofeedback training only modulated the brain connectivity in a local—and not in a global—manner. For this observation, we considered the complex interconnectivity underlying different attention aspects: We considered networks related to externally oriented attention (SAN), internally oriented attention (DMN), executive control and top‐down modulation of attention (FPCN; Dixon et al., 2018; Dosenbach et al., 2006), and switching between external and internal modes of attention (SAL) (Andrews‐Hanna et al., 2014; Seeley et al., 2007). The absence of large‐scale network changes was made for resting‐state runs, that is, when the participants were not explicitly asked to perform the learned ability. We observed positive connectivity between FPCN and DAN and between SAL and DMN independent on the resting‐state session, although the FPCN and the SAL can modulate their coupling with both DAN and DMN depending on the external/internal task nature (Spreng, 2012). The observed positive correlation between FPCN and DAN is likely a consequence that the network definition used privileged a subfraction of the FPCN that is highly coupled with the DAN (Dixon et al., 2018). The observed positive correlation between SAL and DMN might be because of the characteristic mind‐wandering mode adopted at rest (Kucyi et al., 2017; Spreng, 2012).

Sustained attention improved to some extent right after neurofeedback training, but this improvement did not last (Figure 7). In contrast, other studies reported persistent or even increasing behavioral effects following neurofeedback training (Amano et al., 2016; Cortese et al., 2017; Rance et al., 2018; Shibata et al., 2011). However, also other neurofeedback studies found that behavioral effects that were present right after training did not persist. For example, an electroencephalogram‐based neurofeedback study on nicotine addiction reported that short‐term changes in symptom reduction were followed by a gradual return toward the baseline in the long term (Bu et al., 2019). Why neurofeedback training sometimes induces lasting or even improving behavioral effects, while sometimes such effects do not persist, is a crucial question, especially for clinical neurofeedback applications. Here, we can only speculate that, for example, the effect size of the initial behavioral improvement might matter. Our study trained healthy participants in a cognitive domain that we are highly trained in—attention. As a consequence, the behavioral improvement was rather small, possibly due to ceiling effects. This might be different in clinical samples (e.g., Rance et al., 2018). Therefore, studies on follow‐up neurofeedback should, whenever possible, contain information about effect sizes to help elucidate this argument. Another factor might be that for behavioral effects to increase over time, frequent use of learned self‐regulation in everyday situations might be important. Such practice is more likely the case in clinical populations and can be promoted by, for example, electronic diaries (Zaehringer et al., 2019). In general, the association between neurofeedback‐induced brain changes and behavioral effects remains yet to be clarified. For example, Shibata et al. found improved perceptual sensitivity after neurofeedback training even when participants did not actively self‐regulate their visual cortex activity (Shibata et al., 2011), whereas another study found that sensitivity improved only when participants actively up‐regulated visual cortex activity (Scharnowski et al., 2012). In the present study, we found lasting brain changes, but the behavioral effects were only transient. Only the rsFC brain changes showed the same pattern as the behavioral effects: They were present during post‐training runs but no longer during follow‐up runs. Following this temporal coincidence, one might speculate that rsFC changes might serve as a correlate for behavioral effects, but such a speculation requires further investigation.

Also, the relationship between mental strategies and behavioral changes requires further investigation. In our study, the individual choice of mental strategies cannot easily explain that sustained attention improved 1 day after neurofeedback training but no longer during follow‐up 2 months later. Most subjects used the same strategies in both sessions, and most of these strategies were closely related to externally and internally oriented focused attention during regulation and baseline blocks, respectively. It is worth mentioning that, in the follow‐up session, participants were not reminded of the strategies adopted during the initial training. Further, performance during follow‐up transfer runs was not dependent on whether participants used the same strategy right after training or a different one. Thus, learned self‐regulation did not seem to depend on remembering and applying the exact mental strategy that was adopted during training.

### 4.3 Outreach

First, whenever possible, one should include follow‐up neuroimaging assessments of functional and/or anatomical plasticity due to the training rather than only shortly after intervention or only behavioral or regulation‐specific measurements. Although still specific to the MR setting, resting‐state assessments may better reflect transfer effects of neurofeedback training because they are independent of self‐regulation efforts. Follow‐up neuroimaging measurements can help indicate neural reshaping over time after completed interventions (Robineau et al., 2017). If neurofeedback‐induced effects continue to increase over time, measuring them only shortly after a training intervention may lead to undervalued or undetected behavioral effects (Rance et al., 2018). Importantly, follow‐up sessions may help consolidate neuroscientific theories using neurofeedback as a causal intervention (Sulzer, Sitaram et al., 2013) and define biomarkers as targets for neurotherapy (Yamada et al., 2017). Second, since it is desired in a clinical setting that a given intervention converts practice into enduring effects, follow‐up assessments can justify whether the proposed neurofeedback approach is a meaningful alternative for therapy. Therefore, neurofeedback studies that address symptoms should always rely on follow‐up evaluations. Third, we note that, while clear long‐lasting effects in terms of neural self‐regulation may exist, persistent behavioral changes can eventually be dissociated from brain findings (Sitaram et al., 2017). Therefore, follow‐up evaluations of behavioral effects should also be conducted whenever possible. An eventual brain‐behavior dissociation may raise questions about the utility of a proposed neurofeedback approach for modulating behavior or mitigating symptoms in an out‐of‐scanner scenario, the strategic choice of sensitive psychometric instruments, and the characterization of the targeted population. Fourth, we argue that, whenever possible, neurofeedback training and resting‐state/psychometric acquisitions should be made on different days since sleep plays an important role in consolidating learning and producing lasting changes in the brain (Walker & Stickgold, 2004). Indeed, in an extensive neurofeedback training study, Auer and colleagues have shown that performance may improve more over days than over sessions, indicating the consolidating effect of sleep on neurofeedback learning (Auer et al., 2015). Fifth, our study provides evidence that short sessions are sufficient (two training sessions of 45 min on separate days) to produce long‐term effects (Rance et al., 2018) in terms of regulation of brain activity and connectivity changes.

### 4.4 Limitations

The main limitation of this study is that the follow‐up assessments did not include a control group. While our previously reported study included at least a control group that performed the psychometric tasks without neurofeedback training, the present analysis does not include a behavioral nor a neurofeedback control group. For example, a control group that performs neurofeedback regulation of other brain regions or that receives artificially generated feedback would control for spatially non‐specific effects and perception of success (Sorger et al., 2019). Another possibility is that the mere practice of attention without neurofeedback training may have led to the observed neural and behavioral effects. The inclusion of a group that only performs mental‐rehearsal training based on sustained attention, either inside or outside the scanner, would be needed to control for such a possibility. The test–retest (control) group included in our previously reported study only controlled for behavioral practice effects, which do occur in the psychometric tasks that we applied (Langner et al., 2023). Therefore, we cannot conclude with certainty that the observed behavioral and brain changes were caused by neurofeedback training and not by, for example, mental rehearsal inside or outside the scanner. The changes we observed could be due to spontaneous fluctuations over time, habituation to the MR environment, or fatigue, for instance. On the other hand, the fact that the post‐training self‐regulation results were reproducible during follow‐up runs 2 months later and the fact that the brain changes were predominantly specific to the trained brain areas suggest that the brain changes were indeed associated with neurofeedback training. However, to establish with certainty that neurofeedback training causes the behavioral and brain changes that we observed, additional mental‐rehearsal and sham‐control groups would be necessary (Sorger et al., 2019). This was beyond the resources available for this study but should be considered when this approach might be applied clinically.

A second major limitation is the modest sample size. Resource constraints like limited MR scanner availability and scanning costs make scanning larger samples difficult, especially because participants in neurofeedback experiments are being scanned repeatedly. With *N* = 15 and each of these subjects having been scanned on five different days (resulting in a total of 75 MR acquisitions), this study is well within the standard range for fMRI‐based neurofeedback studies (Fede et al., 2020). To accommodate the moderate sample size statistically, non‐parametric tests such as TFCE for statistical mapping in low sample sizes were used. The low sample size also affected the significance tests and a more liberal approach was used for the activation analysis (results not corrected for multiple comparisons, Section 3.1.1.). However, for this analysis, we report Cohen's *d* in the range of 0.46 to 0.64, indicating a moderate effect size and a promising result for future investigations.

Finally, the follow‐up session was acquired 2 months after the end of the training. While 2 months seem sufficiently long to assess lasting effects that go beyond immediate post‐training changes, other studies showed that neurofeedback training effects can last much longer (Amano et al., 2016; Ramot et al., 2017; Robineau et al., 2017; Zilverstand et al., 2015). Hence, from one follow‐up after 2 months, we cannot infer the temporal course and an upper bound for neurofeedback training effects.

## 5 CONCLUSION

The goal of neurofeedback training is to modulate behavior, emotion, cognition, or clinical symptoms in the long term through self‐regulating brain activity. To evaluate whether this ambition has been achieved, follow‐up assessments are key. We found that 2 months after the end of neurofeedback training, participants were still able to exert self‐regulation of the differential SAN‐DMN activity, and this during transfer runs without feedback. Lasting brain changes also included FC measures of the trained ROIs to other brain regions in runs during which participants engaged in active self‐regulation as well as during resting‐state runs without concomitant self‐regulation. These results provide information on important facets of follow‐up assessments: (a) maintenance of the initially learned self‐regulation skill (i.e., SAN‐DMN regulation), (b) maintenance of brain changes related to self‐regulation that go beyond the trained ROIs (i.e., FC changes during transfer runs), and (c) plastic brain changes in the absence of ongoing self‐regulation (i.e., resting‐state changes). Another important aspect of follow‐up assessments is (d) behavioral effects. While others found behavioral effects to increase after neurofeedback training (Rance et al., 2018), the (relatively weak) behavioral effects we observed right after the training did not persist. Such a discrepancy between lasting brain changes, but transient behavioral effects poses important questions regarding the brain–behavior associations above and beyond neurofeedback. Overall, this study highlights the importance of follow‐up investigations of neural and behavioral changes associated with neurofeedback training so that this promising approach can develop its full potential as a scientific and clinical tool.

## CONFLICT OF INTEREST STATEMENT

All authors declare no conflicts of interest.

### PEER REVIEW

The peer review history for this article is available at https://publons.com/publon/10.1002/brb3.3217.

## Supporting information


**FIGURE S1** Target regions of interest (ROIs) for SAN (a) and DMN (b) for neurofeedback training in superior, anterior, and right views.
**FIGURE S2** Learned self‐regulation of the differential SAN‐DMN activity was maintained during both follow‐up transfer runs 2 months after neurofeedback training (a).
**FIGURE S3** Whole‐brain analyses show that the middle and inferior occipital cortex (part of the dorsal attention network) were less activated in post‐training and follow‐up sessions, compared to the pre‐training session.
**FIGURE S4** Whole‐brain maps for the two follow‐up transfer runs analyzed separately.
**FIGURE S5** (a) The regulation‐specific functional connectivity (FC) analyses showed that some clusters presented differences of FC with individual SAN regions across sessions, considering transfer runs.
**TABLE S1** Selected SAN and DMN regions for the neurofeedback training.
**TABLE S2** Resting‐state FC changes within and between canonical large‐scale networks (default mode [DMN], dorsal attention [DAN], frontoparietal control [FPCN], and salience [SAL]).
**TABLE S3** Strategies for regulation and baseline blocks, as well as the concentration scores, reported by each participant during pre‐training, post‐training, and follow‐up transfer runs.

## Data Availability

All obtained results and scripts used for the data analysis are available on the public GitHub repository: https://github.com/gustavopamplona/Followup_NF_attention.
