# Supplementary material for: Preliminary findings on long‐term effects of fMRI neurofeedback training on functional networks involved in sustained attention
Source: Brain Behav. 2023 Aug 18;13(10):e3217. doi: 10.1002/brb3.3217 (PMC10570501; doi:10.1002/brb3.3217)

**Supplementary material**

Long-term effects of fMRI neurofeedback training on functional networks involved in sustained attention

Gustavo S.P. Pamplona^1,2,3,4^, Jennifer Heldner^3^, Robert Langner^5,6^, Yury Koush^7^, Lars Michels^8,9^, Silvio Ionta^1^, Carlos E.G. Salmon^2^*, Frank Scharnowski^3,9,10,11^*

1. Sensory-Motor Laboratory (SeMoLa), Jules-Gonin Eye Hospital/Fondation Asile des Aveugles, Department of Ophthalmology/University of Lausanne, Lausanne, Switzerland
2. InBrain Lab, Department of Physics, University of Sao Paulo, Ribeirao Preto, Brazil
3. Department of Psychiatry, Psychotherapy and Psychosomatics, Psychiatric Hospital, University of Zurich, Switzerland
4. Rehabilitation Engineering Laboratory (RELab), Department of Health Sciences and Technology, ETH Zurich, Zurich, Switzerland
5. Institute of Systems Neuroscience, Heinrich Heine University Dusseldorf, Dusseldorf, Germany
6. Institute of Neuroscience and Medicine, Brain & Behaviour (INM-7), Research Centre Julich, Julich, Germany
7. Department of Radiology and Biomedical Imaging, Yale School of Medicine, Yale University, New Haven, CT, USA
8. Department of Neuroradiology, University Hospital Zurich, Zurich, Switzerland
9. Neuroscience Center Zurich, University of Zurich and Swiss Federal Institute of Technology, Zurich, Switzerland
10. Zurich Center for Integrative Human Physiology (ZIHP), University of Zurich, Zurich, Switzerland
11. Department of Cognition, Emotion, and Methods in Psychology, Faculty of Psychology, University of Vienna, Vienna, Austria

* Both authors contributed equally.

# Figures


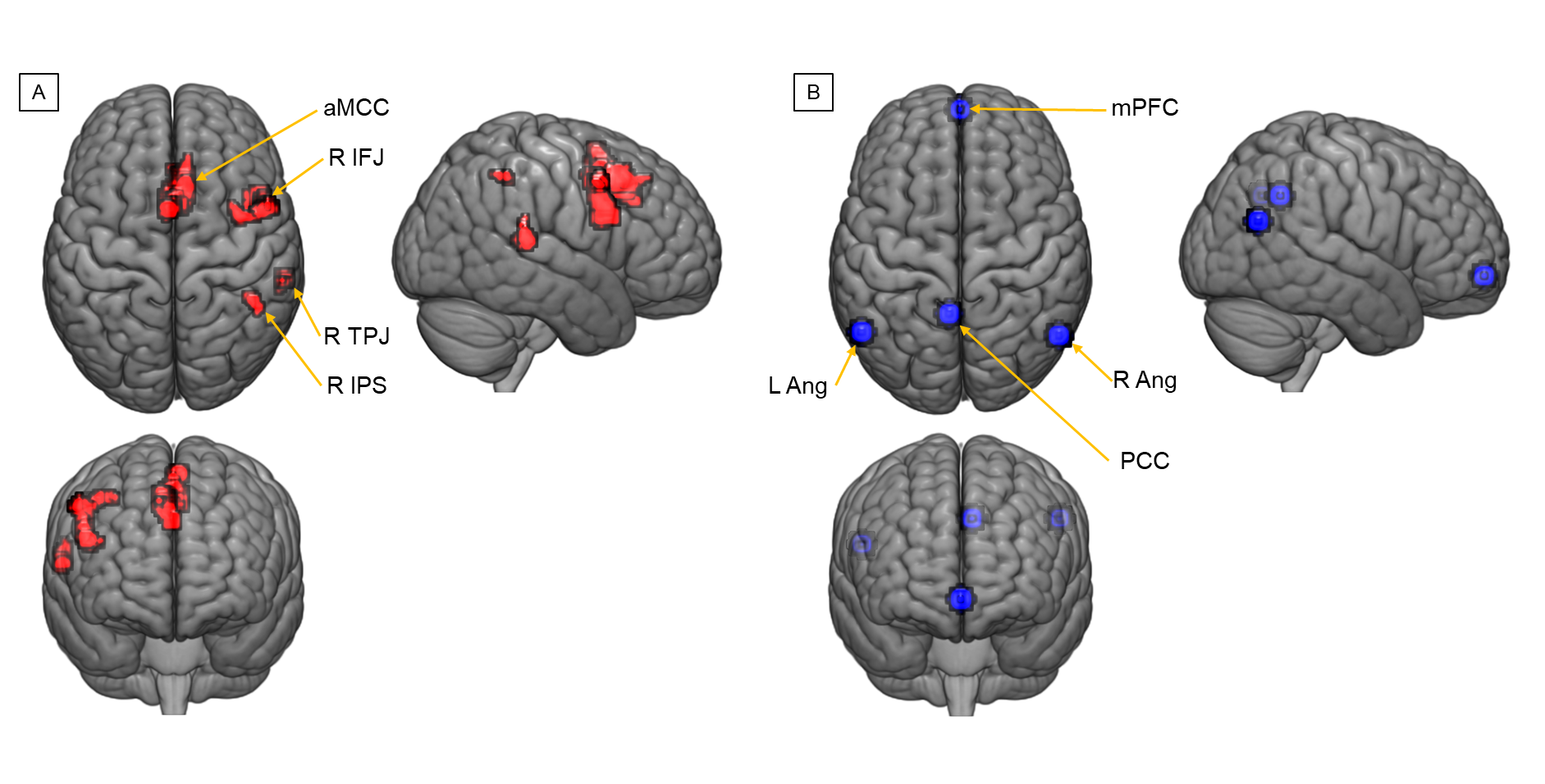


Figure S1. Target regions-of interest (ROIs) for SAN (A) and DMN (B) for neurofeedback training in superior, anterior, and right views. Because the DMN definition was individualized across subjects, the ROIs shown in B are the ones from one of the study’s participants. SAN = sustained attention network, DMN = default mode network, aMCC = anterior midcingulate gyrus, R IFJ = right inferior frontal junction, R TPJ = right temporoparietal junction, R IPS = right intraparietal sulcus, PCC = posterior cingulate cortex, mPFC = medial prefrontal cortex, L Ang = left angular gyrus, R Ang = right angular gyrus.


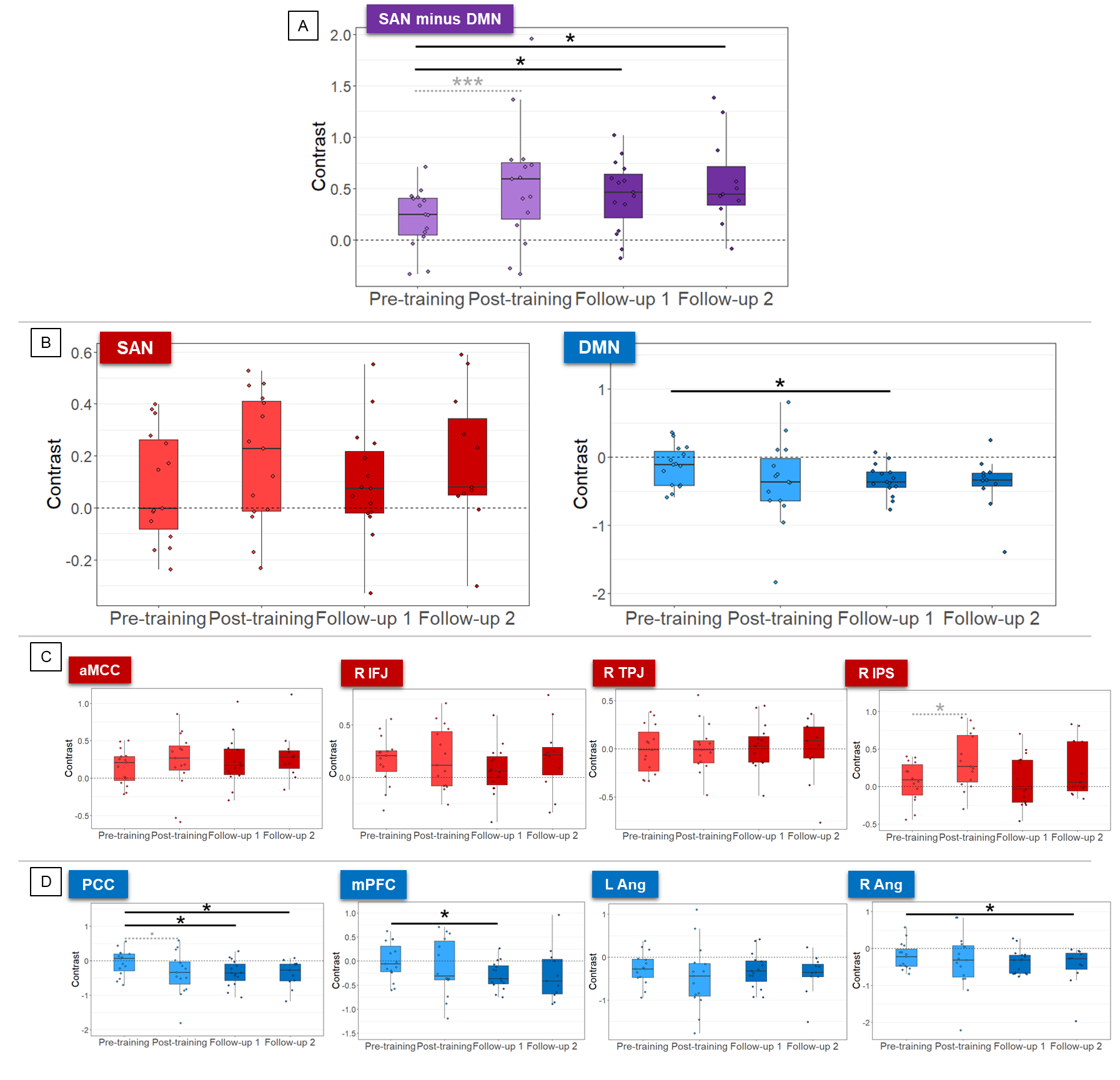


Figure S2. Learned self-regulation of the differential SAN-DMN activity was maintained during both follow-up transfer runs two months after neurofeedback training (A). Self-regulation was mainly driven by down-regulation of the DMN, observed in the first follow-up transfer run (B). No individual regions-of-interest from the SAN showed significant differences across sessions (C). The posterior cingulate cortex (PCC) as part of the DMN showed maintained down-regulation during both follow-up runs (D). Learned down-regulation of the medial prefrontal cortex (mPFC) and right angular gyrus (R Ang) were observed only for first and second follow-up transfer runs, respectively. The graphs show the activation contrast between regulation and baseline blocks for pre-training, post-training, and the two follow-up transfer runs. Purple and blue colors represent the differential SAN-DMN activity and DMN regions, respectively. Light and dark colors represent pre-/post-training and follow-up sessions, respectively. The gray dashed lines represent significant differences previously reported in (Pamplona, Heldner, et al., 2020). Asterisks indicate significant session differences (*** p < 0.001, * p < 0.05, uncorrected). SAN = sustained attention network, DMN = default mode network, aMCC = anterior midcingulate gyrus, R IFJ = right inferior frontal junction, R TPJ = right temporoparietal junction, R IPS = right intraparietal sulcus, L Ang = left angular gyrus.


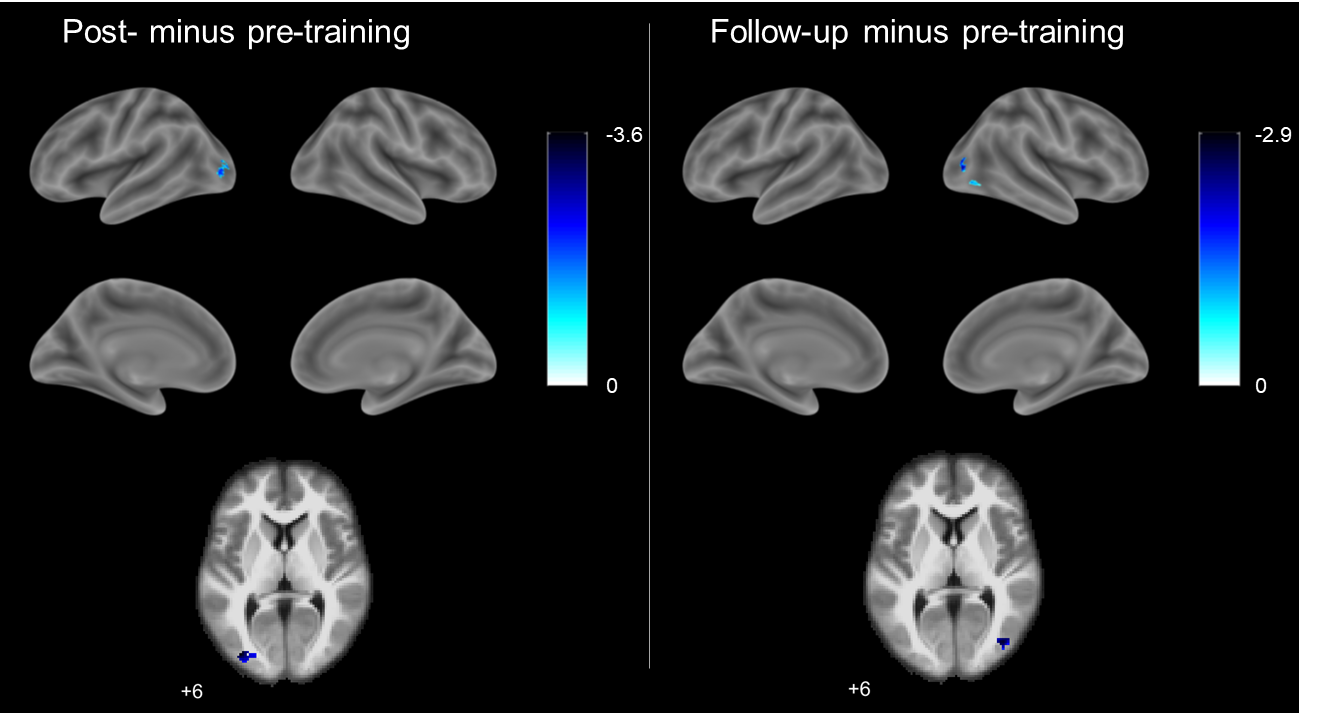


Figure S3. Whole-brain analyses show that the middle and inferior occipital cortex (part of the dorsal attention network) were less activated in post-training and follow-up sessions, compared to the pre-training session. Left and right columns show contrasts post- minus pre-training sessions and follow-up minus pre-training sessions, respectively. Cold colors represent significant negative t-values, respectively, overlapped onto surface-rendered (top) and axial slices (bottom) of a brain template.


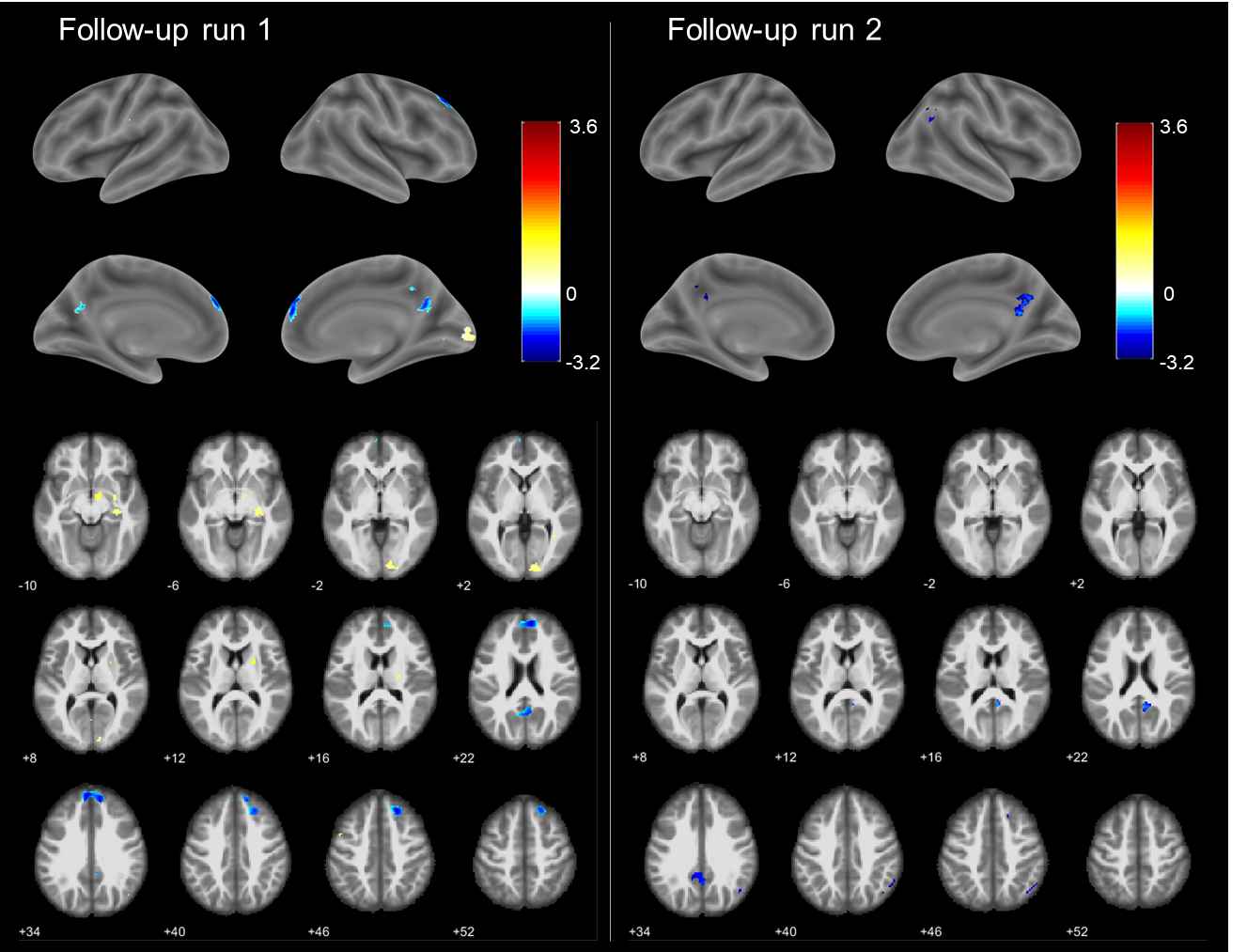


Figure S4. Whole-brain maps for the two follow-up transfer runs analyzed separately. Hot and cold colors represent significant activation and deactivations during regulation compared to baseline blocks, respectively, overlaid on surface-rendered (top) and axial slices (bottom) from a brain template. T-maps were generated by threshold-free cluster enhancement, thresholded at p < 0.001 uncorrected for illustration.


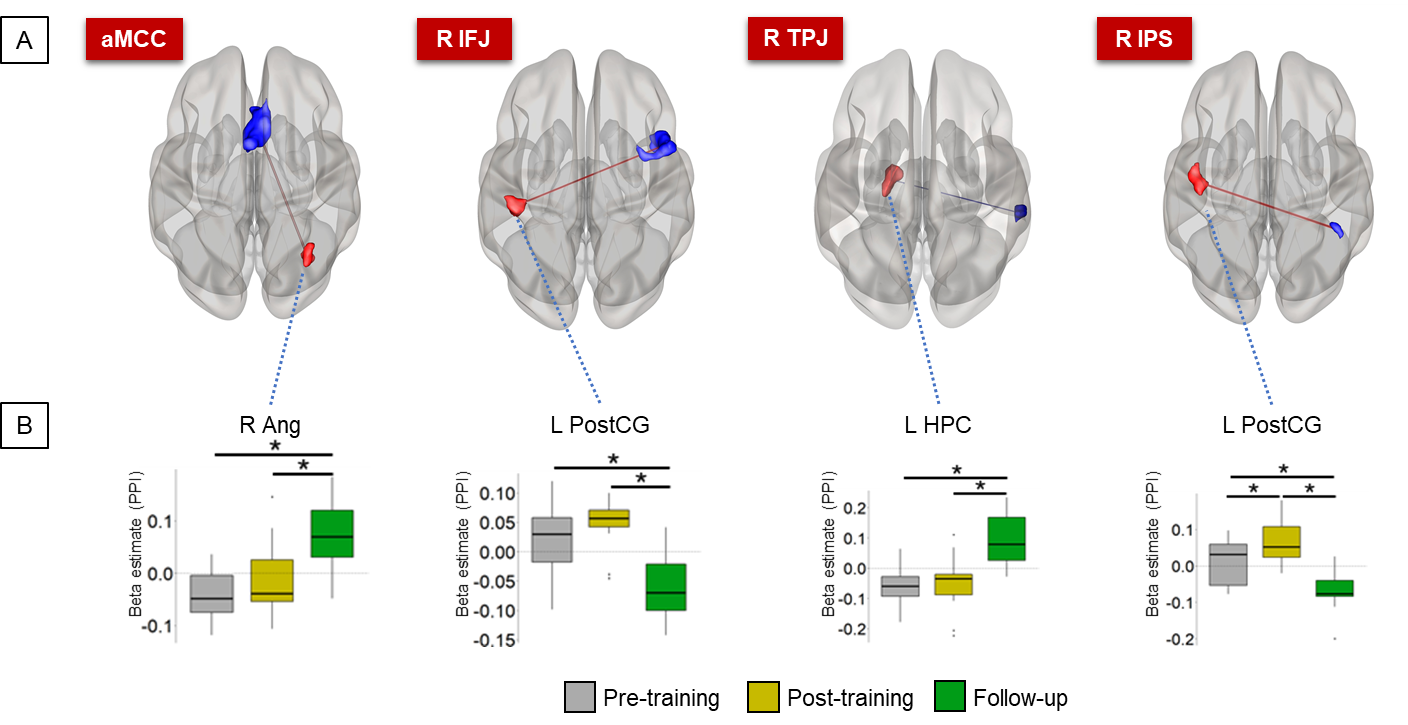


Figure S5. (A) The regulation-specific functional connectivity (FC) analyses showed that some clusters presented differences of FC with individual SAN regions across sessions, considering transfer runs. Blue and red clusters represent SAN regions and seed-to-voxel significant FC clusters, respectively, projected onto glass brains in superior view. (B) Boxplots represent the individual betas estimated for the PPI regressor of the SAN ROIs for each session; gray, yellow, and green represent pre-training, post-training, and follow-up sessions, respectively. The dashed black lines in the boxplots represent the zero level. Asterisks represent significant differences corrected for multiple comparisons by the Tukey method (p < 0.05). SAN = sustained attention network, aMCC = anterior midcingulate gyrus, R IFJ = right inferior frontal junction, R TPJ = right temporoparietal junction, R IPS = right intraparietal sulcus, Ang = angular gyrus, PostCG = postcentral gyrus, HPC = hippocampus, L/R = left/right, PPI = psychophysiological interaction.

# Tables

**Table S1.** Selected SAN and DMN regions for the neurofeedback training.

| Brain region | Number of voxels | MNI coordinates (mm) | | |
| --- | --- | --- | --- | --- |
|  |  | **x** | **y** | **z** |
| Anterior midcingulate cortex (aMCC) | 682 | 2.0 | 14.1 | 47.0 |
| Right inferior frontal junction (R IFJ) | 345 | 46.8 | 7.1 | 34.5 |
| Right temporoparietal junction (R TPJ) | 85 | 60.8 | -33.9 | 13.7 |
| Right intraparietal sulcus (R IPS) | 29 | 43.9 | -45.6 | 46.6 |
| Posterior cingulate cortex (PCC) | 123 | 0 | -54 | 32 |
| Medial prefrontal cortex (mPFC) | 123 | 0 | 52 | 38 |
| Left angular gyrus (L Ang) | 123 | -48 | -64 | 34 |
| Right angular gyrus (R Ang) | 123 | 52 | -62 | 34 |

Note: The SAN regions were the same for all subjects and the clusters were obtained from (Langner and Eickhoff, 2013). The DMN regions were individually defined using resting-state scans and the Personode toolbox (Pamplona et al., 2020b). MNI coordinates for DMN regions are shown as the median across participants on each axis. SAN = sustained attention network, DMN = default mode network, MNI = Montreal Neurological Institute.

**Table S2.** Resting-state functional connectivity (FC) changes within and between canonical large-scale networks (default mode [DMN], dorsal attention [DAN], frontoparietal control [FPCN], and salience [SAL]). Mean and confidence intervals were computed using bootstrapping for FC within clusters of the same network (labeled within) and between clusters of different networks (labeled between), as well as for the differences post-training minus pre-training sessions and follow-up minus pre-training sessions. Asterisks show significant differences (for a significance level of 0.05).

| Networks | Pre-training transfer | Post-training transfer | Follow-up transfer | Post- minus pre-training transfer | Follow-up minus pre-training transfer |
| --- | --- | --- | --- | --- | --- |
| Within-network connectivity | | | | | |
| DAN-DAN | 0.33* [0.29; 0.38] | 0.31* [0.27; 0.35] | 0.33* [0.28; 0.38] | -0.021 [-0.06; 0.02] | -0.004 [-0.05; 0.04] |
| DMN-DMN | 0.56* [0.44; 0.63] | 0.56* [0.49; 0.62] | 0.60* [0.54; 0.66] | -0.004 [-0.09; 0.08] | 0.04 [-0.04; 0.12] |
| FPCN-FPCN | 0.32* [0.29; 0.35] | 0.34* [0.30; 0.38] | 0.33* [0.30; 0.38] | 0.016 [-0.03; 0.06] | 0.010 [-0.03; 0.05] |
| SAL-SAL | 0.30* [0.25; 0.35] | 0.28* [0.23; 0.32] | 0.27* [0.23; 0.31] | -0.028 [-0.10; 0.05] | -0.029 [-0.09; 0.04] |
| Between-network connectivity | | | | | |
| DAN-DMN | -0.20* [-0.26; -0.14] | -0.17* [-0.22; -0.12] | -0.20* [-0.27; -0.13] | 0.028 [-0.04; 0.09] | -0.004 [-0.09; 0.09] |
| DAN-FPCN | 0.12* [0.08; 0.15] | 0.11* [0.07; 0.16] | 0.10* [0.05; 0.14] | -0.0022 [-0.05; 0.04] | -0.015 [-0.05; 0.02] |
| DAN-SAL | -0.024 [-0.06; 0.010] | 0.0020 [-0.03; 0.03] | 0.003 [-0.04; 0.04] | 0.026 [-0.011; 0.06] | 0.027 [-0.013; 0.07] |
| DMN-FPCN | 0.0029 [-0.05; 0.06] | 0.0011 [-0.05; 0.04] | 0.03 [-0.015; 0.08] | -0.0017 [-0.05; 0.04] | 0.03 [-0.03; 0.09] |
| DMN-SAL | 0.13* [0.09; 0.16] | 0.10* [0.04; 0.15] | 0.11* [0.05; 0.17] | -0.029 [-0.07; 0.012] | -0.016 [-0.05; 0.02] |
| FPCN-SAL | -0.015 [-0.05; 0.02] | 0.021 [-0.006; 0.05] | 0.009 [-0.02; 0.04] | 0.04 [-0.002; 0.07] | 0.023 [-0.01; 0.06] |

**Table S3.** Strategies for regulation and baseline blocks, as well as the concentration scores, reported by each participant during pre-training, post-training, and follow-up transfer runs.


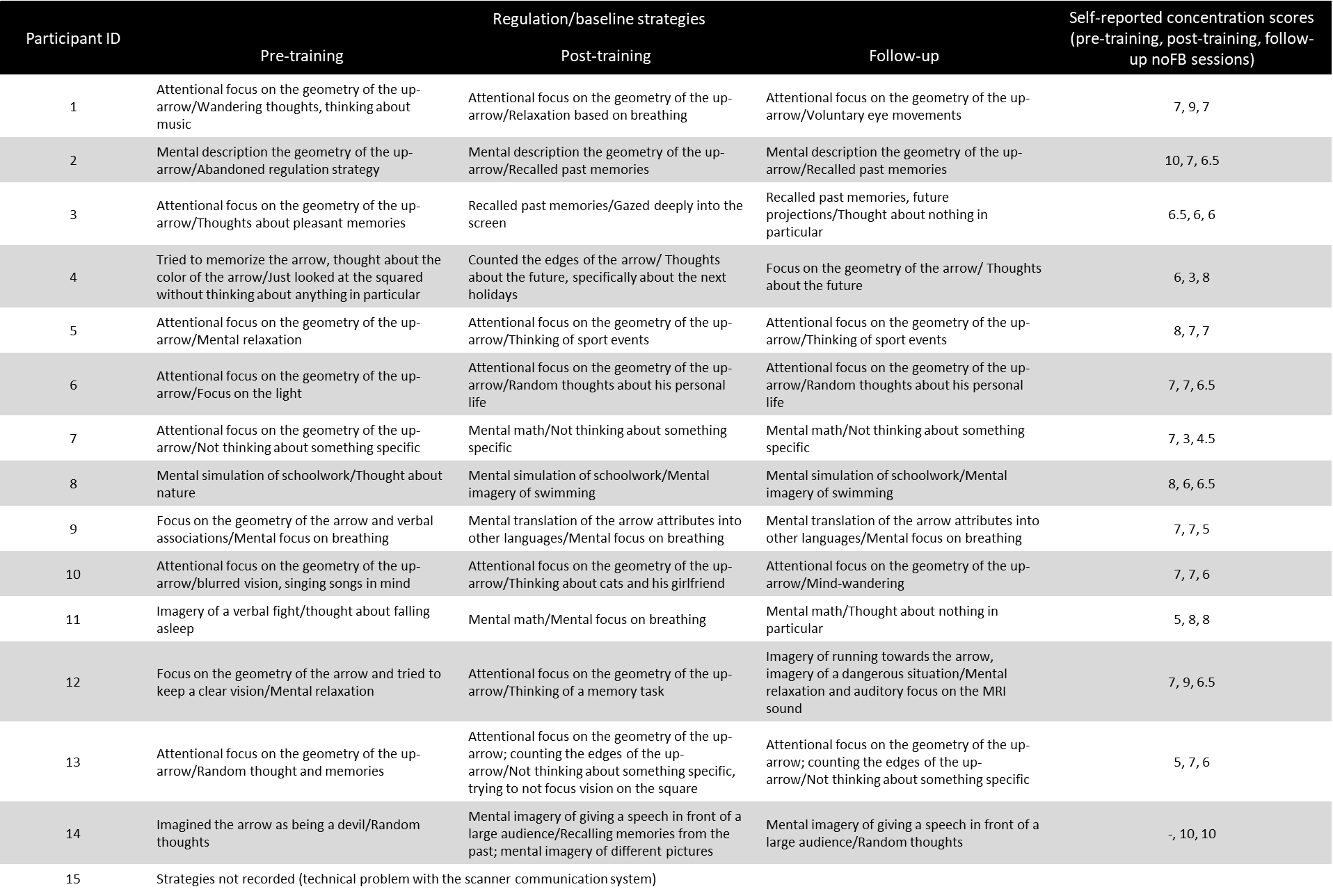

Supplement: Supplementary file 1 — FIGURE S1 Target regions of interest (ROIs) for SAN (a) and DMN (b) for neurofeedback training in superior, anterior, and right views. FIGURE S2 Learned self‐regulation of the differential SAN‐DMN activity was maintained during both follow‐up transfer runs 2 months after neurofeedback training (a). FIGURE S3 Whole‐brain analyses show that the middle and inferior occipital cortex (part of the dorsal attention network) were less activated in post‐training and follow‐up sessions, compared to the pre‐training session. FIGURE S4 Whole‐brain maps for the two follow‐up transfer runs analyzed separately. FIGURE S5 (a) The regulation‐specific functional connectivity (FC) analyses showed that some clusters presented differences of FC with individual SAN regions across sessions, considering transfer runs. TABLE S1 Selected SAN and DMN regions for the neurofeedback training. TABLE S2 Resting‐state FC changes within and between canonical large‐scale networks (default mode [DMN], dorsal attention [DAN], frontoparietal control [FPCN], and salience [SAL]). TABLE S3 Strategies for regulation and baseline blocks, as well as the concentration scores, reported by each participant during pre‐training, post‐training, and follow‐up transfer runs. [file BRB3-13-e3217-s001.docx]
